# Quantitative computerized analysis demonstrates strongly compartmentalized tissue deformation patterns underlying mammalian heart tube formation

**DOI:** 10.7554/eLife.108559

**Published:** 2026-07-21

**Authors:** Morena Raiola, Miquel Sendra Sendra, Jorge N Domínguez, Miguel Torres

**Affiliations:** 1 Cardiovascular Regeneration Program, Centro Nacional de Investigaciones Cardiovasculares (CNIC) Madrid Spain; 2 Department of Experimental Biology, Faculty of Experimental Sciences, University of Jaén Jaén Spain; 3 Centro de Investigación Biomédica en Red de Enfermedades Cardiovasculares (CIBERCV) Madrid Spain; Aix-Marseille Université France; Max Planck Institute for Heart and Lung Research Germany

**Keywords:** heart development, morphogenesis, video microscopy, embryogenesis, Mouse

## Abstract

The quantitative analysis of tissue deformation at cellular resolution remains an important challenge in mammalian organogenesis. Here, we developed a new computational workflow to extract regional and temporal patterns of tissue deformation, and applied it to a collection of live microscopy datasets from mouse cardiogenesis. We devised a method to track tissue deformation directly from time-lapse raw images and experimentally validated the method by comparison with actual cell tracks. We then used a machine-learning approach to temporally and spatially align different specimens and reconstruct a single statistical model of tissue motion, deducing maps of strain, anisotropy, and tissue growth. We also implemented a virtual fate mapping tool that allows tracking any initial position in the cardiac primordium onto the linear heart tube (HT). Our study reveals predominant local cellular coherence during the deformation of the cardiac tissue, whereas strong compartmentalization of tissue deformation patterns transforms the bilateral cardiac primordium into a 3D longitudinal HT. At the future outer curvature of the primitive tube, the ventricular chamber forms by expansion of the tissue in a hemi-barrel shape with two harnessing belts: one that constrains tissue expansion at the arterial pole and one that constrains the expansion at the venous pole. Our study provides a new approach to understanding heart morphogenesis and proposes a new model of primitive HT formation.

## Introduction

Quantifying tissue morphogenesis at the cellular level is essential for understanding developmental mechanisms, addressing congenital heart disease, and designing tissue engineering strategies. In mammals, the heart is the first organ to form and function. Heart morphogenesis is particularly challenging to study because it involves extreme tissue deformation and occurs concurrently with the onset of cardiac beating. Early cardiogenesis involves extensive reorganization and progressive differentiation of the cardiogenic fields to rapidly create a pumping structure (Ivanovitch et al., 2017; Kelly et al., 2014; Meilhac et al., 2014; Buckingham et al., 2005; Moorman and Christoffels, 2003). In mice, the first differentiation wave produces a simple single layer known as the cardiac crescent (CC). The CC then undergoes a series of deep deformations, gradually forming the heart tube (HT), a sophisticated three-dimensional structure with inflow (IFT) and outflow (OFT) tracts and the ability to support embryonic circulation (de Boer et al., 2012). While the evolution of tissue shape during cardiogenesis has been characterized and quantitatively analyzed (Ebrahimi et al., 2022; Esteban et al., 2022; Kawahira et al., 2020; Le Garrec et al., 2013), understanding how tissues deform during morphogenesis requires the definition of cell displacement and rearrangement patterns.

Recent advances in microscopy and genetic cell labeling have opened new avenues for investigating tissue dynamics. Live-imaging techniques, enabling non-invasive observation of biological phenomena, have opened new opportunities and perspectives in this field (Dominguez et al., 2023; Raiola et al., 2023; Sendra et al., 2022; Ivanovitch et al., 2017; Le Garrec et al., 2017). These new approaches have provided access to datasets that include tissue deformation and cellular tracks; however, these datasets remain underexploited for global, multiscale analysis of tissue deformation during cardiogenesis.

While some studies have focused on the dynamic analysis of discrete morphogenetic events of early heart development (Sendra et al., 2025; Ivanovitch et al., 2017; Buckingham et al., 2005; Meilhac et al., 2004), the global dynamic analysis of tissue and cell dynamics during mammalian heart development has not been achieved. A thorough understanding of the morphogenetic mechanisms of early heart development requires adopting a multiscale approach that incorporates the behavior of individual cells, but at the same time describes the global dynamics at the tissue level.

Here, we address this challenge by applying a previously developed and validated computational workflow, described in detail in Raiola et al., 2025, to quantify and compare tissue deformation patterns by integrating motion profiles estimated from multiple 3D live fluorescence microscopy datasets. The workflow is based on a non-invasive analysis of individual live images and quantifies tissue kinematics to compute deformation fields and principal strain directions, without modeling the forces underlying such motion. To align the individual motion profiles of multiple specimens over time, the workflow incorporates a staging system that synchronizes 3D live images with a previously described pseudo-dynamic Atlas of tissue geometry during heart morphogenesis (Esteban et al., 2022). This strategy allows the deduction of deformation patterns with spatial and temporal resolution, capturing both instantaneous features and their cumulative history. In this study, we use this framework to extract and interpret biologically meaningful deformation patterns during heart development. By applying this established workflow to heart development, we report new essential features of cardiogenic tissue deformation, including a strong spatiotemporal compartmentalization of cell behaviors. The 3D+t model generated also enables in silico fate map analysis at the cellular or regional level, which provides a tool to interrogate specific features of the dynamics of cardiac cells during HT morphogenesis. This model lays the groundwork for future predictive simulations of the forces driving heart morphogenesis.

## Results

### Estimating myocardial motion and deformation from multiple time-lapse datasets

**Figure 1.**
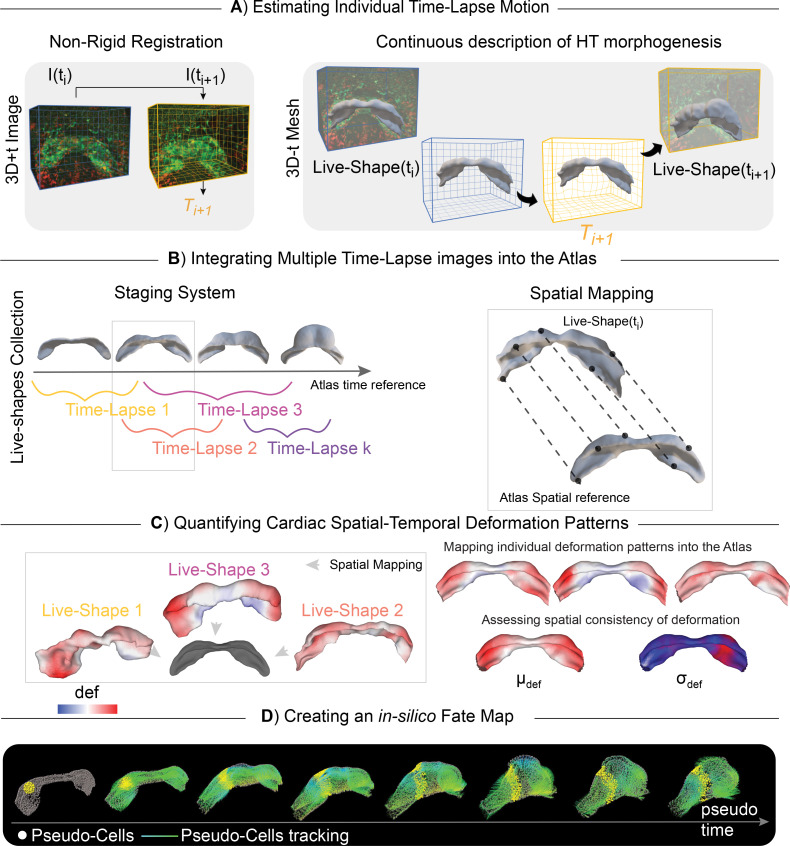
Workflow for extracting and defining the motion and deformation pattern of the myocardium during early heart morphogenesis. The analysis pipeline consists of four main steps. Our dataset includes 16 specimens ranging from E7.75 to E8.25 (12 hr). (**A**) Estimating individual time-lapse motion: A non-rigid algorithm extracts the transformation *T* needed to deform one frame, *I*(*t_i_*), into the next frame, *I*(*t*_*i*+1_). The transformation is computed for a set of control points of a regular 3D grid (blue grid). Control point displacement is updated (orange grid) until the two frames overlap. We collect the set of transformations {*T_i_*} for each time-lapse image to compute the continuous description of heart tube (HT) morphogenesis. (**B**) Integrating multiple time-lapse images into the Atlas: We incorporate the individual continuous descriptions of HT deformation into the Atlas. We define a staging system that aligns the individual time-lapse images on a common time reference and registers the related 3D mesh (Live-Shape) onto the spatial reference (Atlas). (**C**) Quantifying cardiac spatial–temporal deformation patterns: We extract the deformation patterns from each continuous description of HT deformation and compile them into the Atlas, generating a single stage-by-stage model (*μ*_def_, *σ*_def_). (**D**) Creating an in silico fate map: We concatenate a continuous description of HT deformation from the individual steps to recreate a unique and continuous description of early HT deformation patterns. Gray spots represent the initial positions of the pseudo-cells, while color lines represent their displacement during development. Yellow spots indicate the tracked cells.

We applied a workflow that consists of four steps summarized in Figure 1: (A) Individual time-lapse analysis to capture the geometry of the myocardium from the CC to the linear HT; (B) Integration of multiple time-lapse datasets through spatiotemporal registration into a previously described 3D+t Atlas (Esteban et al., 2022); (C) Quantification of cardiac spatiotemporal deformation patterns during CC to HT morphogenesis; and (D) In silico fate mapping to investigate how different CC regions contribute to the forming HT. The full methodology is detailed in a co-submitted manuscript (Raiola et al., 2025).

We analyzed both published (Ivanovitch et al., 2017) and new datasets consisting of 3D+t live images (Raiola et al., 2025), including 16 embryos from early CC formation to the onset of heart looping. Myocardial cells were labeled using various reporter strategies, including Nkx2.5GFP (Wu et al., 2006) or a combination of Nkx2.5Cre (Stanley et al., 2004) with different fluorescent reporter alleles. To enable individual cell tracking, Nkx2.5GFP embryos carried sparse genetic labels induced by low-dose tamoxifen using the RERT allele (CreERT2 knocked-in under the control of RNApol2 promoter; Guerra et al., 2003); these labels were used exclusively as fiducial markers for validation, not for quantitative single-cell analysis. Additional embryos expressed Mesp1Cre to mark all mesodermal cells, or Islet1Cre to label cardiac progenitor populations, including second heart field (SHF)-derived regions. Importantly, no lineage-specific or strain-dependent biological comparisons are performed in this study; fluorescent labeling is used solely to visualize and track tissue motion. Acquisitions were performed at a variety of temporal windows and with variable time-lapse periods. The procedures developed therefore cope with a diversity of labeling strategies, temporal rates, and embryonic periods of acquisition (Raiola et al., 2025).

To estimate tissue motion, we applied the Medical Image Registration Toolbox (MIRT) (Myronenko and Song, 2010), which calculates displacement tensors across time on raw images (Figure 1A; Raiola et al., 2025; Myronenko and Song, 2010). This algorithm produces Tensor vectors (*T*) that describe the displacement of each image point over time (Figure 1A, orange grid). To validate these computational predictions, we compared them to manual tracking of >9000 genetically labeled cells derived from 9 embryos through consecutive frames (Raiola et al., 2025). The validation metric was based on the distance between predicted and experimentally measured positions. The results show that the predicted displacements closely match real cell movements, with an average error at the subcellular level, confirming that MIRT provides high-resolution and accurate quantification of tissue deformation during HT formation.

To generate a unified model of cardiac tissue motion, we registered each time-lapse sequence to the 3D+t Atlas of heart development (Esteban et al., 2022). First, we segmented the myocardial tissue in each specimen of the time-lapse datasets and produced a dense mesh of triangles that describes the tissue surface (Raiola et al., 2025). These meshes describe the dynamics of myocardial tissue motion and were named ‘Live-Shapes’. Second, we applied a machine learning-based staging system that uses morphometric features to align live images of different specimens over time, allowing the assignment of each specific specimen and stage to the corresponding Atlas geometry (Figure 1B; Raiola et al., 2025). Third, we mapped specimens onto the Atlas to address shape variability between equally staged hearts (Figure 1B; Raiola et al., 2025).

These steps allowed the mapping of parameters, such as strain and growth, from individual specimens onto the Atlas, providing their distribution and regional variability among specimens (Figure 1C, example of tissue growth mapping). Finally, using the tissue deformation data, we performed in silico fate mapping by simulating the forward displacement of pseudo-cells placed in the CC domain. This analysis showed how different CC regions contribute to specific domains of the forming HT and how they deform during this process (Figure 1D).

### Extracting deformation patterns from the continuous description of HT morphogenesis

**Figure 2.**
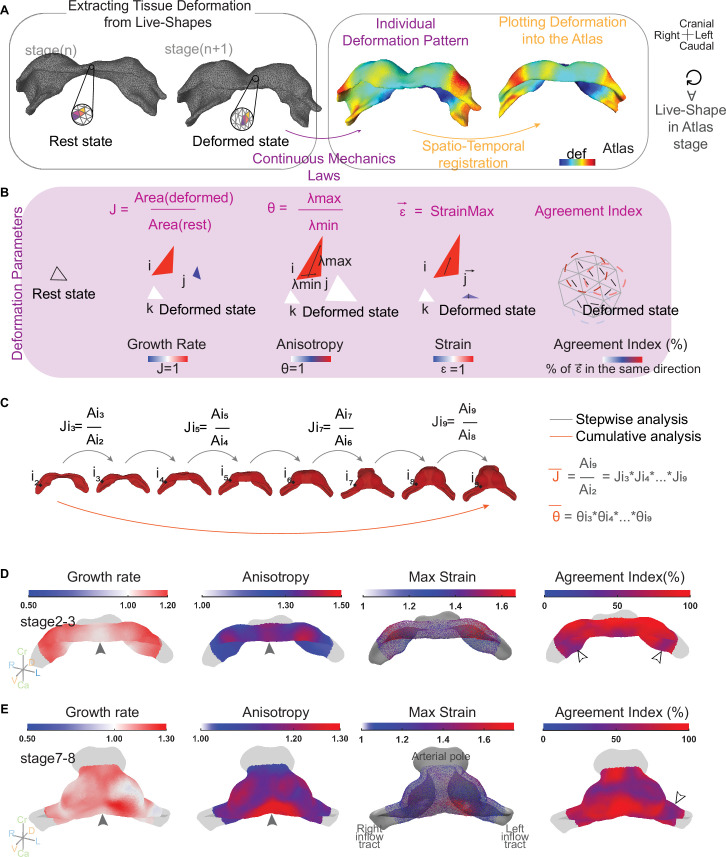
Deformation pattern analysis. (**A**) Mesh transformation from the initial state to the deformed state. The triangles of the mesh are transformed according to the MIRT tensor from time *t_i_* to time *t_i_*_+1_. The deformation pattern is calculated by applying the principles of continuum mechanics between the initial and deformed states. This pattern is then visualized on the deformed shape. The spatiotemporal registration onto the Atlas enables the mapping of the deformation into it. (**B**) Description of the deformation parameters between the initial and deformed states. For each triangle, three different possible deformations (a, b, c) are represented, color-coded according to the parameter value. The growth rate (*J*) is defined as the ratio of the area of the same triangle before and after deformation. Anisotropy (*θ*) is defined as the ratio between the maximum and minimum deformation magnitudes. The maximum strain (*ε*) is characterized by the vector of maximum deformation. The agreement index reports the local coherence in the direction of tissue deformation. (**C**) Difference between stepwise deformation pattern analysis (gray arrows) and cumulative deformation pattern analysis (orange arrows). (**D, E**) Stepwise deformation pattern analysis in caudal view. The color maps represent the growth rate, anisotropy, strain magnitude, and direction, and finally, their agreement. Here, the deformation maps are shown for the transition from stage 2 to stage 3 and from stage 7 to stage 8. The full arrowhead indicates a zone of low growth rate and high anisotropy in the most ventral-medial region. The empty arrowhead indicates discontinuity in the agreement of the strain direction. Deformation values are the mean values. The number of specimens averaged at stages 2–3 is 5, while at stages 7–8 it is 2.

To analyze tissue deformation patterns in the collection of Live-Shapes, we applied the continuous mechanics laws to the deformation of the mesh triangles (Figure 2A, Raiola et al., 2025). Deformation was calculated between consecutive time points for 11 embryos, each with at least two shapes classified in successive Atlas stages. A subset of embryos was excluded because a substantial portion of the IFT was lost due to embryo drift, ensuring that all regions of the cardiac tissue are represented by the same number of embryos in the cumulative deformation analysis. The resulting pattern was then mapped onto the following stage shape, capturing the immediate deformation history. For each triangle, we computed local shape changes and applied smoothing (Raiola et al., 2025) to estimate local tissue growth rate (*J*), tissue anisotropy (*θ*), and the magnitude and direction of tissue deformation (*ε*) (Figure 2B). In addition, we introduced a new parameter; ‘Strain Agreement Index’ (*φ*), which quantifies local coordination of strain directions. This metric distinguishes regions of coordinated deformation (ordered) from areas with locally discrepant deformation directions (chaotic). It would also identify borders between regions with coherent but discrepant strain directions (Figure 2B, Materials and methods—Strain Agreement Index). All deformation measurements are invariant to translation and rotation, making them robust to embryo drift during acquisition.

**Figure 2—figure supplement 1.**
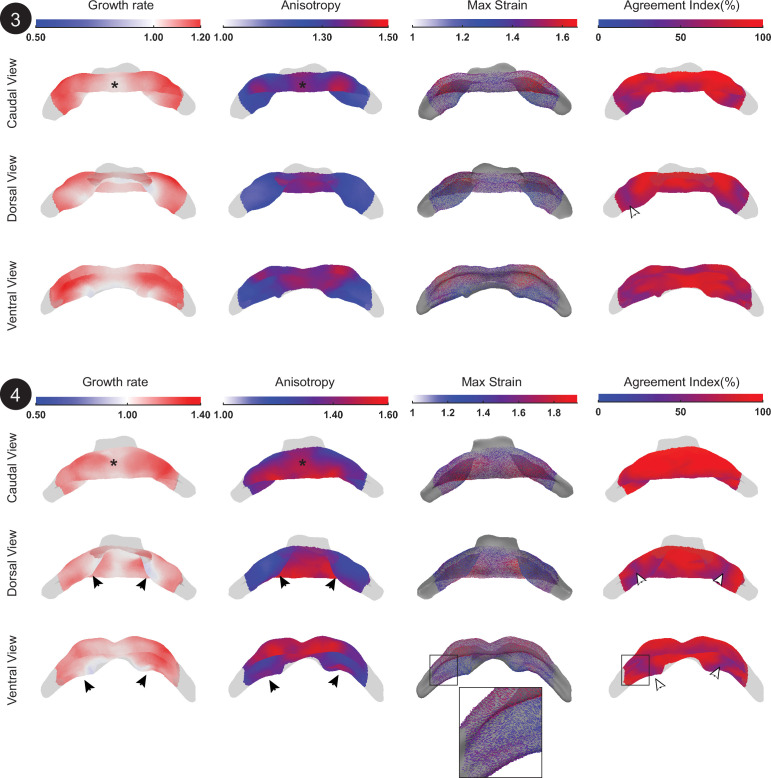
Stepwise deformation pattern analysis. Deformation analysis for stages 3 and 4. The number of specimens averaged at stage 2 is 5, while at stage 4 it is 6.

**Figure 2—figure supplement 2.**
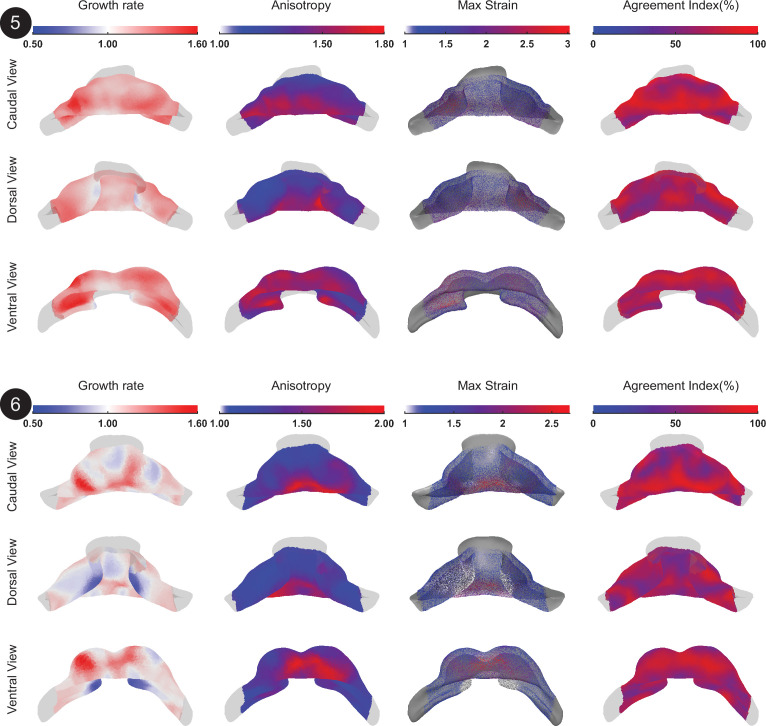
Stepwise deformation pattern analysis. Deformation analysis for stages 5 and 6. The number of specimens averaged at stage 5 is 2, while at stage 6 it is 2.

**Figure 2—figure supplement 3.**
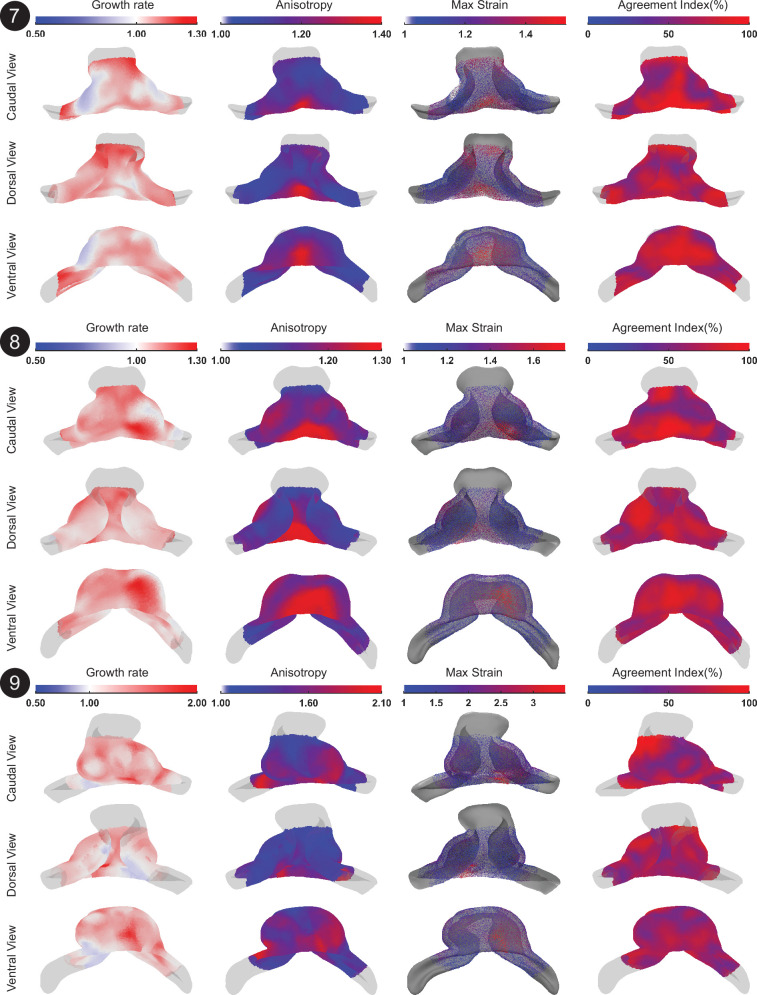
Stepwise deformation pattern analysis. Deformation analysis for stages 7–9. The number of specimens averaged at stage 7 is 1, at stage 8 is 2, and at stage 9 is 1.

**Figure 2—figure supplement 4.**
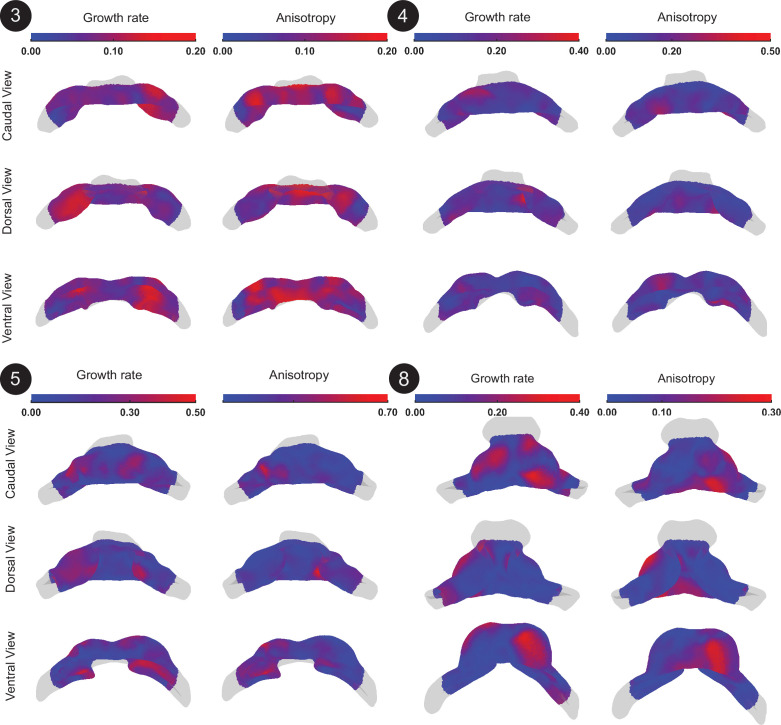
Standard deviation of the growth rate and the anisotropy patterns. Color maps indicate the spatial distribution of the standard deviations for the growth rate and anisotropy magnitudes shown from ventral, dorsal, and caudal views. Stages 6, 7, and 9 have only one staged Live-Shape, and therefore variability could not be measured.

**Figure 2—figure supplement 5.**
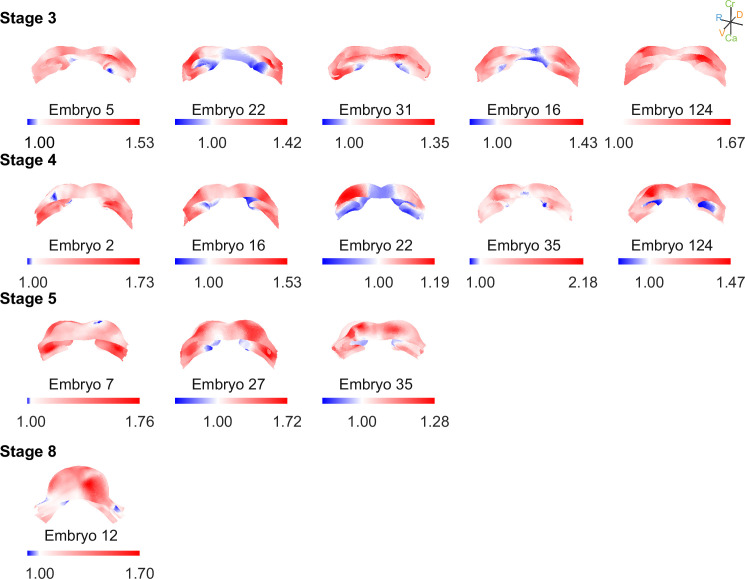
Mean growth rate derived from stepwise deformations between consecutive developmental stages for individual embryos, mapped onto the Atlas geometry (caudal view). Values represent superficial tissue changes, with values <1 indicating compression, values = 1 isochoric deformation, and values >1 expansion. Partial IFTs and the arterial pole are not present in the Atlas. Developmental stages represented by more than one embryo are included.

**Figure 2—figure supplement 6.**
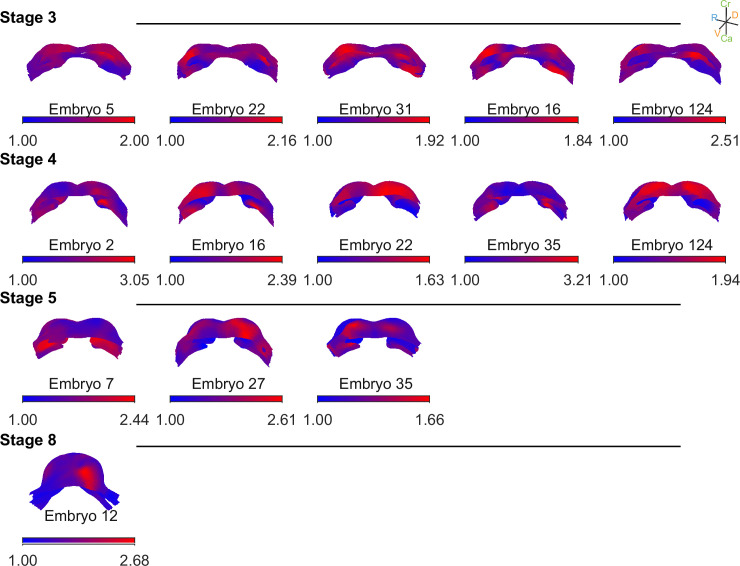
Mean anisotropy rate derived from stepwise deformations between consecutive developmental stages for individual embryos, mapped onto the Atlas geometry (caudal view). Values represent superficial tissue changes, with values = 1 isotropic deformation, and values >1 anisotropic. Partial IFTs and the arterial pole are not present in the Atlas. Developmental stages represented by more than one embryo are included.

We then examined myocardial deformation pattern in two complementary ways: stepwise, by comparing deformation between consecutive stages; and cumulatively, by integrating the stepwise deformations through several stages (Figure 2C). To specifically focus on HT morphogenesis driven by tissue deformation, we excluded Atlas stage 1, given that between stages 1 and 2 substantial cell addition to the HT through myocardial differentiation takes place (Esteban et al., 2022).

### Stepwise deformation pattern analysis

To investigate the spatial consistency of the deformation patterns among specimens, we mapped each deformation value from the Live-Shapes onto the corresponding Atlas geometry. For each stage group, we computed the spatial distributions of the mean deformation (*μ*_def_) (Figure 2—figure supplement 1, Figure 2—figure supplement 2, Figure 2—figure supplement 3) and its variability (*σ*_def_) (Figure 2—figure supplement 4), with Figure 2—figure supplements 5 and 6 showing the individual embryo contributions to these mean and std values. Importantly, while inter-embryo variability reflects natural biological diversity (Figure 2—figure supplement 4), it remained highly localized, primarily affecting deformation magnitude rather than spatial patterns, with anticorrelated growth/anisotropy zones consistently preserved across specimens (Figure 2—figure supplement 5, Figure 2—figure supplement 6).

The stage 2-to-stage 3 and stage 3-to-stage 4 deformation analyses showed an inverse distribution of high growth and high anisotropy regions. High growth was associated with low anisotropy, and high anisotropy appeared in regions with low growth. High anisotropy/low growth was mainly detected in a coherent region spanning the ventral midline region of the HT and extending laterally to the ventrocaudal region adjacent to the peripheral splanchnic mesoderm (see solid arrowheads in Figure 2D, and asterisks in Figure 2—figure supplement 1, Growth and Anisotropy columns). A second high anisotropy/low growth region appeared at the dorsal lips of the HT (see solid arrowheads in Figure 2—figure supplement 1, Growth and Anisotropy columns). The rest of the HT, including the IFTs, showed a largely isotropic growth (Figure 2—figure supplement 1). In stage 4, a left-biased increase in growth was detected (Figure 2—figure supplement 1, Growth column). Most specimens at this stage presented a coherent deformation direction, with strain oriented along the craniocaudal axis. Accordingly, they generally showed a high Agreement Index, indicating coordinated deformation of the myocardium. An exception to this is found at the lateral parts of the IFTs, where narrow bands of local strain disagreement were found separating the medial from the lateral parts of the IFTs (see empty arrowheads in the Agreement column). A closer observation of the strain alignment in these regions (see magnifications in the Max Strain column of Figure 2—figure supplement 1) shows that the low-agreement bands correspond to an abrupt transition between regions of coherent but discrepant strain directions. Whereas in the lateral-most regions of the IFTs strain aligns parallel to the main IFT axis, in their proximal region strain aligns craniocaudally, coherently with the rest of the HT (Figure 2—figure supplement 1, Max Strain and Agreement columns). These results indicate a strong compartmentalization of collective cell behavior during HT morphogenesis. This compartmentalization affects both the anisotropy/growth patterns and the directionality of tissue deformation. Furthermore, this analysis detected a strong anticorrelation between tissue growth and anisotropic deformation.

In stages 5 and 6, the medio-caudal myocardium continued to deform anisotropically along the craniocaudal direction, with a high rate of agreement, indicating coordinated deformation (Figure 2—figure supplement 2). At this stage, high growth rates are localized to the ventrolateral regions and extend into the inflow regions from the lateral regions of the forming ventricle (Figure 2—figure supplement 2). In general, high growth rates are mostly present in the caudal regions of the myocardium, except for some medio-lateral areas of the forming ventricle at stage 6 (Figure 2—figure supplement 2). This is accompanied by the appearance of regions of low agreement in the bilateral bulging areas of the forming ventricle (Figure 2—figure supplement 2). With some exceptions, growth and anisotropy continued to show anticorrelated patterns (Figure 2—figure supplement 2). The inflow regions again showed sharp boundaries between regions of high agreement with discrepant directions, indicating high compartmentalization of the deformation directions.

In stages 7 and 8, the caudal region of the myocardium showed reduced growth, whereas new high-growth areas appeared in the ventromedial region of the forming ventricle, extending bilaterally toward the cranial region around the arterial pole (Figure 2E, Figure 2—figure supplement 3). In all regions of the forming ventricle, growth correlated with a high index of agreement and, in general, with low anisotropy (Figure 2—figure supplement 3). The inflows continued showing frontiers of strong disagreement separating the distal inflow, predominantly showing a medio-lateral deformation direction, from more proximal regions, showing craniocaudal deformation directions (Figure 2—figure supplement 3). In stage 8, a left-side-specific increase in growth was observed in the ventral side of the ventricle.

In stage 9, the most prominent feature was the rightward rotation of the ventricle (Figure 2—figure supplement 3), a phenomenon related to heart looping (Le Garrec et al., 2017). This was accompanied by a sharp increase in growth and decrease in anisotropy at the left junction between the IFT and the ventricle, whereas the opposite took place on the right side (Figure 2—figure supplement 3). As in stage 8, higher growth was also observed in the ventral left side of the ventricle, suggesting its relationship to ventricle rotation.

An interesting observation throughout this study is the behavior of the myocardial rim in contact with the splanchnic mesoderm at the prospective dorsal pericardial wall. This border of the CC deforms drastically toward the cranial pole to form the rim of the arterial pole and the dorsal myocardial lips that fuse forming the mesocardium. This complex deformation is essential for the transformation of the CC into primitive HT. The dorsal and ventral views of the myocardium from stage 3 to stage 6 (Figure 2—figure supplements 1 and 2) show that this rim is divided into two regions with different behaviors: a medial region that coincides with the closing dorsal lips and deforms toward the cranial pole, and bilateral distal regions, remaining at their original position. Interestingly, the medial aspects of this rim show very reduced growth or even contraction, whereas its lateral aspects show high growth rates. These results thus show again a strong compartmentalization of growth and deformation during HT formation.

### Cumulative deformation pattern analysis

**Figure 3.**
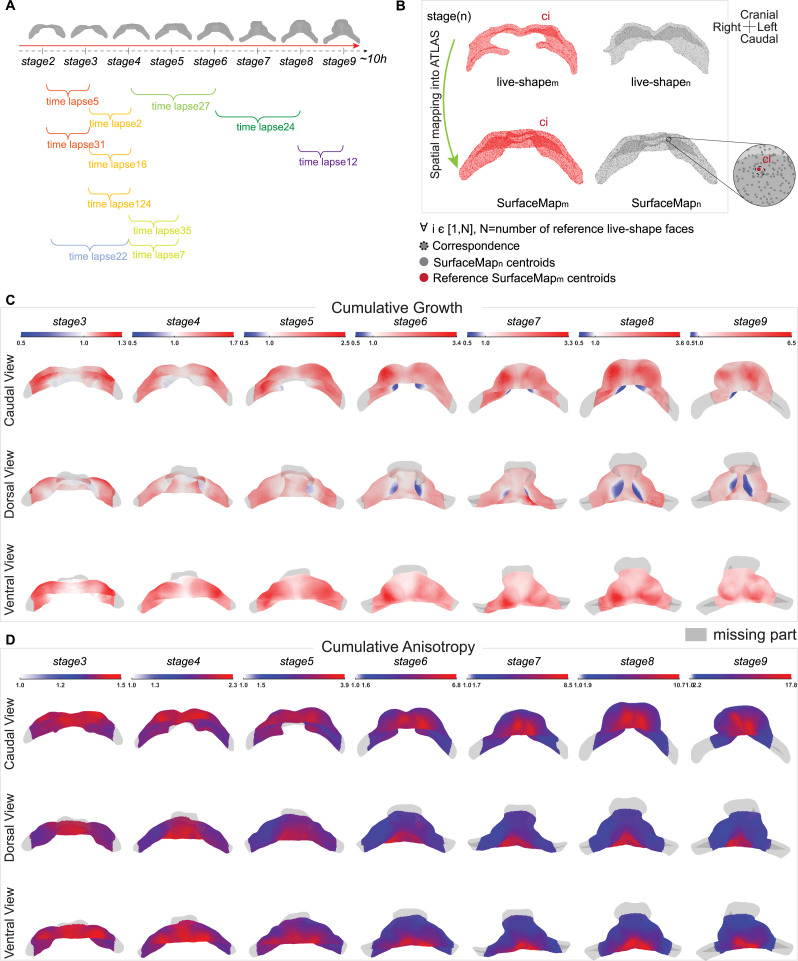
Cumulative deformation pattern analysis. (**A**) Concatenation of multiple time-lapses on a common timeline. Each time-lapse covers sub-windows of the Atlas temporal line. (**B**) Schematic description of the concatenation pipeline. We fix a reference sample and for each equally staged sample, we select the centroids of the SurfaceMap closest to the reference SurfaceMap. (**C**) Cumulative growth of heart tube (HT). The color map refers to the mean growth rate of the tissue from stage 2. (**D**) Cumulative anisotropy of HT. The color pattern indicates which zone of the myocardium accumulates more anisotropic deformation. The color map refers to the mean accumulated anisotropy of the tissue from stage 2. The color bars indicate the deformation magnitude. Gray zones correspond to the missing IFTs and arterial pole parts.

To determine the accumulated changes through several stages, we applied an additional strategy to analyze tissue deformation in chosen time windows (Raiola et al., 2025). The purpose was to recreate the continuous motion profile of the heart tissue during its transformation from CC to HT, exploiting the extracted kinetic information from individual live-imaging sequences. This strategy concatenates the motion profiles derived from the live images to fully cover from stage 2 to stage 9, allowing the cumulative deformation analysis (Raiola et al., 2025; Figure 3A).

We used the projections of the Live-Shapes onto the Atlas, which we called ‘SurfaceMap’ (Figure 3B), to establish correspondence between specimens and concatenate their motion profiles (Raiola et al., 2025). In this way, we could map the accumulated growth from stage 2 through the different stages. We found that the highest growth accumulated in the IFTs and at bilateral ventrolateral areas of the forming ventricle, whereas the midline and the arterial pole remained at low growth rates (Figure 3C). The two ventricular bilateral growth regions appeared as focal areas, with a clear single center from which growth rates decline concentrically (Figure 3C, ventral view). These high-growth foci progressively displace medially during development but always keeping bilaterality up to stage 9. The rim of the myocardium in contact with the prospective dorsal pericardium again showed two distinct regions; a bilateral distal region of relatively high growth in the IFTs, and the region of the dorsal closure, which shows low growth (Figure 3C). In contrast, anisotropic deformation accumulated along the ventral midline and the dorsal closure region of the myocardial rim, whereas it remained low in the expanding regions of the tissue (Figure 3D). These results confirm the strong compartmentalization of growth and deformation, with growth predominating in lateral regions, and deformation along the craniocaudal direction in medial regions.

### In silico fate map analysis and its use in determining tissue motion during HT morphogenesis

**Figure 4.**
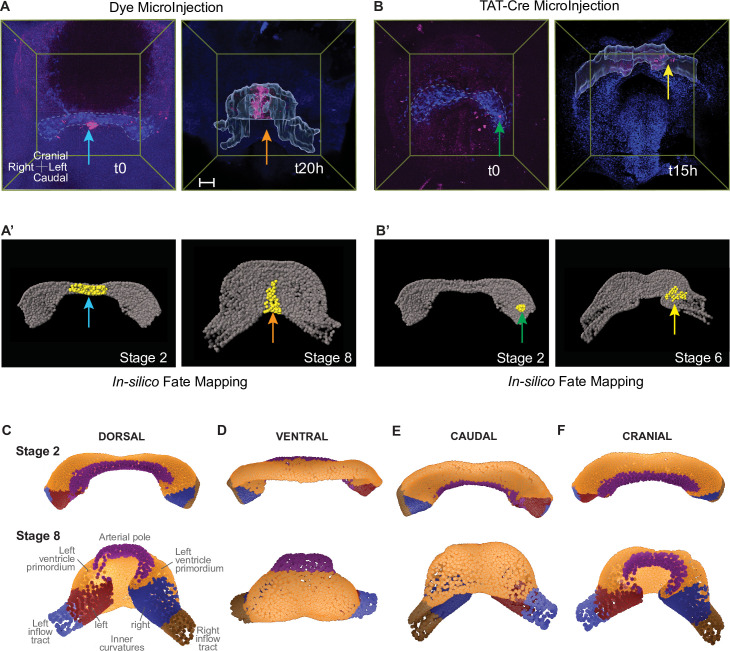
In silico fate map. (**A**) Labeling of a cardiac crescent (CC) discrete region by dye injection (t0, left) and 3D reconstruction of its contribution to the heart tube (HT) after 15 hr of embryo culture (t15h, right). (**A’**) Labeling on stage 2 virtual model of an equivalent region to that experimentally labeled in (**A**) (left) and the resulting labeled region in the stage 6 virtual model (right). (**B**) Labeling of a CC discrete region (t0, left) by TAT-Cre-induced recombination and 3D reconstruction of its contribution to the HT after 20 hr of embryo culture (t20, right). (**B’**) labeling on stage 2 virtual model of an equivalent region to that experimentally labeled in (**A**) (left) and the resulting labeled region in stage 8 virtual model (right). (**C–F**) different views of the virtual model showing different regions of the HT in stage 8 and their primordia in stage 2. Scale bar: 15 µm.

Next, we used the concatenation strategy to create a deterministic in silico fate map reproducing the motion profile of the myocardium during early morphogenesis. This strategy involved displacing the reference SurfaceMap nodes in accordance with the motion profile, generating a ‘Dynamic Atlas’ as described in Raiola et al., 2025. In this Dynamic Atlas, every vertex of the model can be tracked from stage 2 to stage 9 (see Figure 4—video 1). Given the correspondence between the model and the actual cell displacement (Raiola et al., 2025), the resulting in silico fate map can be interpreted in terms of pseudo-cellular displacement within the myocardium. We next validated the Dynamic Atlas by developing the tool ‘fate map’, which allows us to label any position/s and determine its fate in silico. At the regional level, we compared the predicted regional growth in the Dynamic Atlas with that directly measured from the ex vivo labeled cultured embryo. We performed experiments on ex vivo cultured embryos using the TAT-Cre injection (Sendra et al., 2023) and dye labeling (Domínguez et al., 2012) to experimentally determine the fate of specific CC regions (Supplementary file 1). Using the TAT-Cre technique, we labeled a discrete group of cells in the medial CC and tracked the positions of the cell progeny after culturing the embryo for 20 hr (Figure 4A). An equivalent label generated in the stage 2 virtual model generated a labeled region in the stage 8 virtual model similar to that obtained in the experimental setting (Figure 4A, A’). Next, we labeled a group of embryonic cells with dye and examined the fate of the label after 15 hr (Figure 4B). We then labeled equivalent positions in the stage 2 virtual model and found that the fate of this region in the stage 6 virtual model was similar to that obtained in the experimental setting (Figure 4B, B’).

**Figure 4—video 1.** In silico fate map. The Dynamic Atlas is represented in its point-cloud version. At the cellular level, a yellow spot tracks one pseudo cell position throughout heart morphogenesis. At the tissue level, yellow spots illustrate the tissue deformation occurring during heart morphogenesis.

Once determined that the virtual model approximates the behavior of myocardial cells during HT formation, we used it to map the primordia of the different regions of the HT observable at stage 8 (Figure 4C–F). We labeled the arterial pole, the left ventricle primordium (outer curvature), the ventricular side of the future inner curvature (dorsal side of the HT opposing the forming ventricle), and the IFTs. The comparison between the primordia and the end-stage structures indicates that the lateral-most parts of the CC grow and extensively migrate medially to generate the IFTs and the dorsal part of the HT (future inner curvature), whereas the primordia of the future ventricle (outer curvature) and the arterial pole mainly undergo deformation to achieve their final shape. Whereas both the arterial pole and the future ventricle reshape toward the midline, the arterial pole region contracts to a cylindrical shape, while the future ventricle bulges out in a barrel shape (Figure 4C–F).

**Figure 5.**
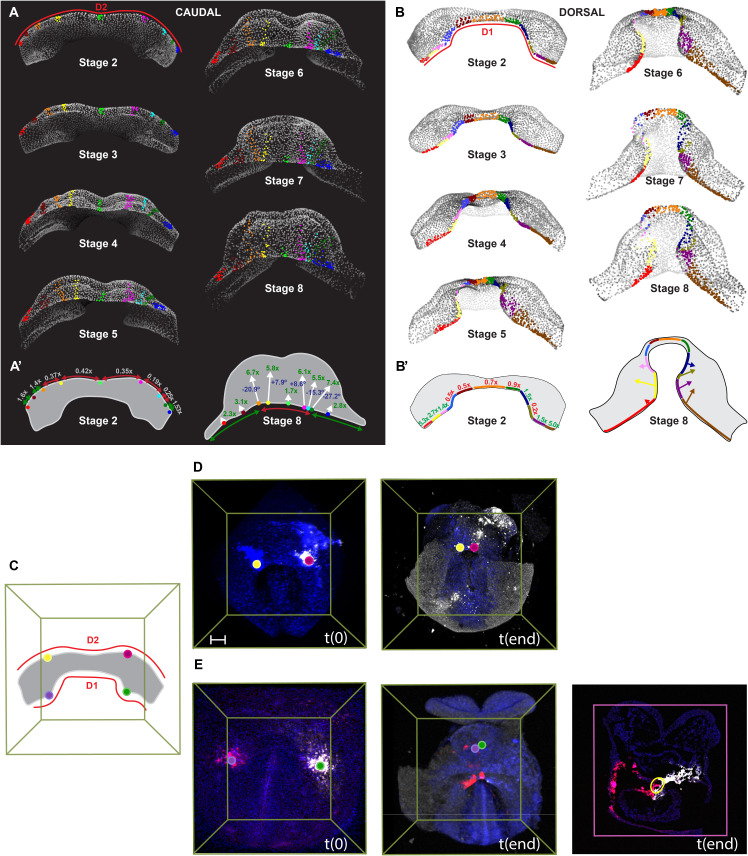
Fate maps of the cardiac crescent (CC) boundaries reveal tissue dynamics of heart tube (HT) formation. (**A**) Clusters of pseudo-cells were labeled at regular spacing along the D2 line of the stage 2 CC model. The labeled clusters were then followed through stage 8. Caudal views are shown, which allow us to visualize the caudal side of the developing inflows and ventricle. (**A’**) Left, representation of the degree of expansion (green) or contraction (red) of the different D2 segments defined between the labeled clusters at stage 2. Numbers indicate fold change from the initial to the final time points. Right, representation of the stage 8 view indicating the regions of D2 that contract (red) versus those that expand (green). Fold change cranial ward expansion of each labeled cluster from their original craniocaudal extension is shown in green. The direction of the white arrows indicates the main direction of expansion. The angle of the main expansion direction with respect to the embryo mid-plane is indicated in blue. (**B**) Contiguous segments of pseudo-cells were labeled at regular spacing along the D1 line of the stage 2 CC model. The labeled clusters were then followed through stage 8. Dorsal views are shown. (**B’**) Left, representation of the labeled segments on stage 2 CC with indication of their fold change expansion (green) or contraction (red). Right, representation of the final extension at stage 9 of the D1 labeled segments. Arrows indicate the direction and extension of the expansion of the labeled pseudo-cells to colonize the dorsal parts of the HT. (**C**) Schematic representation of the CC at stage 2 indicating the four anchor points used for experimental validation: two along D2 (yellow and magenta dots) and two along D1 (purple and green dots). (**D**) Experimental validation of D2 contraction. An embryo (e6_D2) was microinjected at the D2 anchor points (yellow and magenta) and imaged by multiphoton microscopy at t0 (left) and after 10 hr (right). The Euclidean distance between the two labeled anchor points was measured at both time points (right image). (**E**) Experimental validation of D1 contraction. An embryo (e1_D1) was microinjected at the D1 anchor points (purple and green) and imaged at t0 (left) and after 10 hr (center). Given the difficulty of imaging the arterial pole at high resolution by whole-mount microscopy, cryosections were performed (right, pink frame) to measure the geodesic distance between anchor points in a coronal plane (arterial pole in yellow). Multiphoton images were acquired at a voxel size of 0.57 × 0.57 × 2.5–6.0 µm. Cryosection images were acquired at a pixel size of 0.65 × 0.65 × 0.042 µm. Scale bar: 15 µm.

Next, to understand how the ventricular barrel shape is produced, we used the virtual model to label groups of cells at regular spacing along the rims between the splanchnic mesoderm and the CC at stage 2 (Figure 5). These two rims define two lines, one between the CC and the juxta-cardiac field (Figure 5A; D2, as described in Esteban et al., 2022) and another between the CC and the SHF (Figure 5B; D1, as described in Esteban et al., 2022).

This study shows that along D2 only the lateral-most sections expand toward the midline to generate the sections of D2 underlying the IFTs at stage 8 (Figure 5A, A’). In contrast, all D2 sections underlying the ventricle at stage 8 undergo a strong compression from their initial length at stage 2 (Figure 5A, A’). Whereas the labels that end up positioned within the IFTs show limited cranial expansion, those underlying the ventricle extensively expand cranially and colonize the caudal part of the ventricle (Figure 5A, A’). At stage 8, the future left ventricle shows two hemi bulges with an indentation between them corresponding to the midline. The strongest lateral compression and cranial expansion are found for bilateral groups of cells close to the midline of these two hemi bulges, while the midline label expands only moderately toward the ventricular wall (Figure 5A, A’). Interestingly, the cranial extensions of these bilateral cell groups open laterally as they extend cranially, generating a fan-like label (Figure 5A, A’). These observations suggest that the lateral constriction and cranial expansion of cells close to D2 contribute to generating the barrel shape of the ventricle.

The same study on D1 shows that the region fated to the arterial pole compresses to form the cylindrical shape of this structure, whereas the lateral aspects of D1 expand drastically to form the cranial border of the IFTs and the dorsal borders of the ventricular region, which will later fuse to form the mesocardium (Figure 5B, B’). In this future mesocardial region, cells originally positioned close to D1 expand to populate the inner curvature of the ventricular region (Figure 5B, B’). These deformations ensure that the HT forms by generating the inner curvature regions and allowing its dorsal closure. At the same time, the constriction imposed by the formation of the arterial pole limits the expansion of the ventricle at its cranial end, thereby contributing to its barrel shape.

To experimentally validate the tissue dynamics predicted by the virtual model, we identified four anchor points, two along D1 and two along D2, to track changes in these boundaries during HT formation (Figure 5C). For D2, three embryos were microinjected at the defined anchor points, imaged around stage 2, and re-imaged after 10–14 hr by multiphoton microscopy (Figure 5D). The Euclidean distance between the two D2 anchor points was computed at t0 and tend. In all three embryos, the anchor points converged over time, with the D2 segment retaining on average 0.27 ± 0.14 of its initial length (range: 0.13–0.40), reflecting a substantial contraction whose magnitude varied depending on the developmental stage and exact position of microinjection. Consistent with these measurements, the model predicted a contraction of approximately 0.38 between the yellow and magenta reference points (Figure 5A’), in agreement with the experimentally observed deformation at the corresponding locations in Figure 5D, E. For D1, three additional embryos were microinjected in the same manner and the in-plane geodesic distance between anchor points was measured at t0 and after 10–14 hr (Figure 5E). Given the difficulty of imaging the arterial pole at high resolution by whole-mount microscopy, cryosections were used. The D1 segment defining the arterial pole similarly underwent compression, retaining on average 0.50 ± 0.22 of its initial length (range: 0.23–0.77). In all cases, the reduction in both D1 and D2 distances over time is consistent with the contraction dynamics predicted by the barrel model (Supplementary file 2).

**Figure 6.**
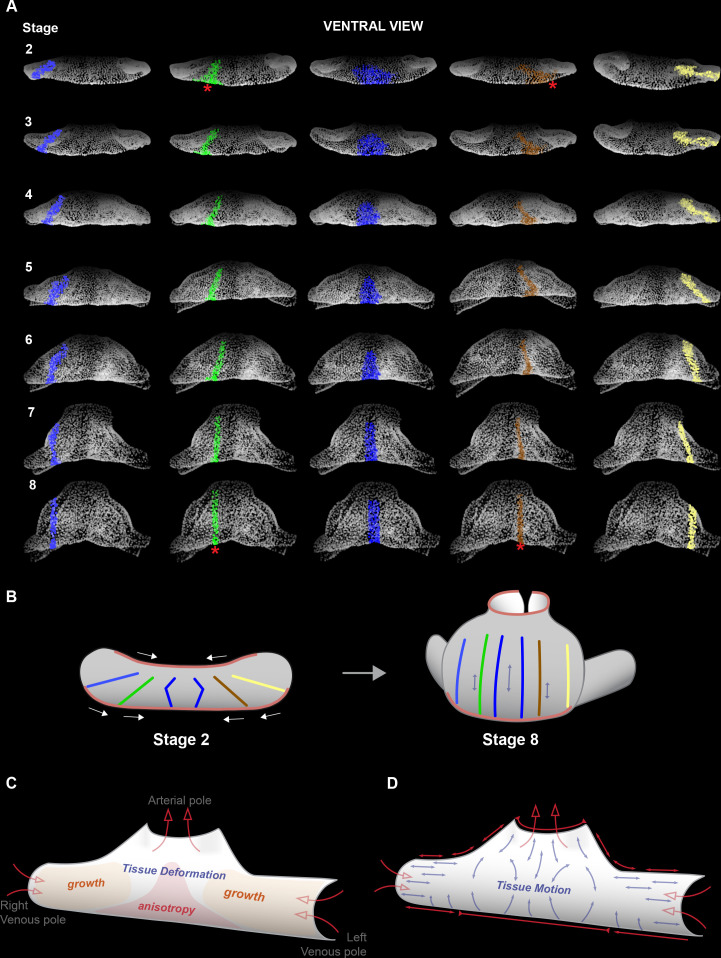
Fate map of the ventricle primordium shows the tissue dynamics underlying ventricle formation. (**A**) Longitudinal rectangular groups of pseudo-cells were regularly labeled in the ventricle of the stage 8 model. The labeled regions were then tracked back through stage 2. (**B**) Schematic representation of regional deformations at stages 2 and 8. (**C**) Representation of the main growth and anisotropy areas in the myocardium. (**D**) Representation of the main directions of tissue motion during heart tube formation.

Finally, to understand the shape changes taking place during the formation of the primitive left ventricle, we labeled longitudinal rectangles in the ventricle of the stage-8 model and studied the evolution of their shape from stage 2 to stage 8 (Figure 6A). The observations indicate that the whole ventricular primordium undergoes extensive reshaping with progressive constriction of the tissue toward the midline in a craniocaudal gradient. The movement resembles the closing of an open fan with the vertex at the cranial side (Figure 6B). Through this movement, lateral tissue is recruited toward the midline and extends cranially. An exception to this general reshaping is the mid-central region of the ventricle, where bilateral deformation takes place in mirror-image directions with an equatorial axis of symmetry. This deformation pattern correlates with the localized fan-like distribution of the descendant of D2 (Figure 5A, A’, indicated by asterisks in Figure 6). These observations therefore match the deformations observed for labels in D1 and D2 (Figure 5). Together, these results show that the ventricle primordium is narrower at the cranial end of the CC and extends progressively more laterally toward the caudal end of the CC. Ventricle morphogenesis thus involves tissue constriction by two ‘belts’ of tissue that support the barrel shape of the ventricle. The cranial belt constitutes the arterial pole and the caudal belt coincides with the myocardial region abutting the juxta-cardiac field. This caudal belt undergoes a much more extensive constriction than the cranial one, related to the recruitment of very lateral aspects of the CC. In contrast, at the cranial aspect of the CC, this constriction is less strong and ventricle formation does not involve the recruitment of the lateral-most CC regions.

## Discussion

Here, we applied a previously established image-based pipeline (Raiola et al., 2025) to extract and compare myocardial deformation during HT morphogenesis in the mouse embryo. Our analysis comprises 16 specimens that have been staged into 9 different canonical shapes at nominal ages ranging from E7.75 to E8.25 (approximately 12 hr). The canonical shapes provide a spatiotemporal reference for comparing and compiling the motion and deformation of multiple specimens.

The knowledge of the deformation patterns extracted from the videos allowed the cumulative evaluation of the most important transformations of the myocardium, measuring their intensity and direction, and determining their timing. The maps reveal a strong compartmentalization of growth rates and anisotropic deformation. Growth is generally high in the lateral regions of the forming ventricle, with a bias toward its caudal parts. In contrast, the medial stripe of the CC, later of the forming tube, does not appreciably grow but shows high-anisotropic deformation with stretching mainly oriented in the rostro-caudal direction. Similar mutual exclusion of the high-anisotropic areas and the growing bilateral caudal regions is clearly maintained until stage 6. This compartmentalization generates clear boundaries between regions that show coordinated stretch directions and those that do not and between regions showing different stretching directions (Figure 6C). These boundaries are particularly pronounced in the inflow region, where differences in stretching directions become particularly evident (Figure 6D). In the IFT, boundary regions suggest two forces that act in different directions: one, stretching the IFT cells in the latero-medial direction, and another promoting the elongation of the tube in a craniocaudal direction. On the dorsal side of the forming tube, the rim that connects the myocardium to the splanchnic mesoderm (D1) also shows a high medio-lateral compartmentalization, with its medial part undergoing little growth or even compression at several stages, whereas its lateral parts grow and stretch toward the midline (Figure 6D). This originates a progressive cranial looping of the medial aspects of this rim, which form the arterial pole and the closing mesocardium, whereas the lateral-most parts remain as IFTs.

To directly test whether the compression dynamics predicted by the virtual model occur in vivo, we performed microinjection experiments tracking anchor points along D1 and D2 over 10–14 hr (Figure 5C–E, Supplementary file 2). In all six embryos analyzed, anchor points converged over time along both boundaries, representing to our knowledge the first direct in vivo evidence that the medial sections of D1 and D2 undergo tissue contraction during HT formation. This observation indirectly implies the expansion of their lateral sections, provided both D1 and D2 expand during development, with D1 showing a much stronger expansion. The variability observed across embryos reflects the inherent challenges of this experimental approach: the exact position of the anchor points could not be perfectly standardized, embryos were not always captured at exactly the same developmental stage at t0 and tfinal, and high-resolution geodesic staging was deliberately avoided to preserve embryo viability. Nonetheless, the consistent direction of shrinking among specimens supports barrel model predictions.

Given that tissue compression inhibits proliferation through the activation of the Hippo pathway (von Gise et al., 2012), we propose that one of the factors contributing to the lower growth of the medial regions might be tissue compression. Alternatively, or in addition, it is possible that forces arise from the splanchnic mesoderm, which is highly proliferative (de Boer et al., 2012) and could contribute to the anisotropic deformation of the mesocardial rim of the myocardium.

Based on these findings, we propose a model for mouse primitive HT morphogenesis in which areas of growth are predominantly lateral in the CC and later become ventrocaudal during the formation of the ventricle. Given that the lateral extension of the heart remains stable during HT formation (Esteban et al., 2022), we propose that the growth of these lateral regions causes the medial convergence of the tissue. This convergence is driven by two belts that constrain the CC toward the midline, a cranial one that coincides with the arterial pole and a caudal one at the boundary between the ventricle primordium and the juxta-cardiac field (Figure 6D). In between these two belt-like constraints, the ventricle adopts a bulging barrel shape (Figure 6B). Surprisingly, ventricle morphogenesis at this stage does not involve localized areas of growth but rather extensive remodeling to recruit lateral tissue and relocate it along the craniocaudal direction (Figure 6B). The model thus proposes complementary regions in which growth and deformation are anticorrelated. Concomitant with this recruitment to medial regions, HT tissue is deformed in the craniocaudal directions, so that the cranial and caudal belts move apart from each other and the HT adopts its longitudinal disposition (Figure 6B).

Between stages 7 and 9, we observed a difference in growth rate between the two sides of the forming ventricle, consistent with de Boer et al., 2012. This bias could be the first input for the looping process. Increased growth in the left region, as observed in the chicken model, could influence the direction of cardiac looping and result in a rightward C-shape (Kidokoro et al., 2008). As a consequence of the slight bending, the tissue could undergo compression on the right side. Alternatively, the observed bias could derive from differential growth of myocardial cells during looping (Ebrahimi et al., 2022; Shi et al., 2014). In addition, differential L–R proliferation in the dorsal pericardial wall may also influence heart looping by influencing the dorsal myocardial rim (Desgrange et al., 2020).

By concatenating the motion profiles of multiple specimens, we present the first in silico fate map, which enables virtual analyses of the dynamics of HT formation. The resulting Dynamic Atlas is a deterministic, descriptive representation of tissue morphogenesis, obtained by integrating individually derived motion fields within a common reference frame. This baseline is essential for studying heart defects, as deviations from normal tissue motion and deformation patterns can reveal developmental defects like altered growth or wrong motion paths. Due to its deterministic nature, the framework does not allow predictions of system responses to perturbations, but it provides a robust baseline for characterizing HT tissue dynamics. This approach reveals strong anisotropic deformation of the tissue, particularly in the ventricle and the dorsal myocardial boundary, where cumulative deformation analysis indicates the greatest anisotropy. Although the accuracy of the kinetic motion captured by the fate map has not been directly validated, we believe it can reliably track cardiomyocyte movement in these regions, as confirmed by comparison with the video-microscopy dataset (Raiola et al., 2025). We propose the Dynamic Atlas and its associated tool, the ‘fate map’, as a valuable approach for exploring HT morphogenesis.

In conclusion, by applying a previously established workflow (Raiola et al., 2025), we provide detailed kinetics of myocardial motion and the tissue deformations underlying early mammalian heart development. The fate map is proposed as an innovative tool to shed light on regionalized cell coordination, generating new questions about the biological and genetic factors that regulate the cardiac development process.

## Materials and methods

**Key resources table:** 

Reagent type (species) or resource	Designation	Source or reference	Identifiers	Additional information
Strain, strain background (*Mus musculus*, male and female)	Polr2a–CreERT2 (RERT)	Guerra et al., 2003	MGI ID: 3772332 RRID:IMSR_JAX:017585	
Strain, strain background (*Mus musculus*, male and female)	Nkx2.5-Cre	Stanley et al., 2004	MGI ID: 2448972 RRID:IMSR_JAX:024637	
Strain, strain background (*Mus musculus*, male and female)	Mesp1-Cre	Lescroart et al., 2014	MGI ID: 2176467	
Strain, strain background (*Mus musculus*, male and female)	Islet1-Cre	Cai et al., 2003	MGI ID: 3053746 RRID:MSR_JAX:024242	
Strain, strain background (*Mus musculus*, male and female)	ROSA26CAG–TdTomato	Madisen et al., 2010	MGI ID: 3809524 RRID:IMSR_JAX:007914	
Strain, strain background (*Mus musculus*, male and female)	ROSA26CAG–EGFP	Sousa et al., 2009	MGI ID: 4412373 RRID:MMRRC_032037-JAX	
Strain, strain background (*Mus musculus*, male and female)	Tg(CBF:H2BVenus,+)	Nowotschin et al., 2013	MGI ID: 5487911 RRID:IMSR_JAX:020942	
Strain, strain background (*Mus musculus*, male and female)	Tg(H2B:miRFP703,+)	Gu et al., 2018	MGI ID: 6383974 RRID:IMSR_JAX:034720	
Chemical compound, drug	TAT-Cre recombinase	Sigma-Aldrich	SCR508	
Chemical compound, drug	Sucrose	Sigma-Aldrich	S0389	
Chemical compound, drug	Ethyl alcohol	Sigma-Aldrich	E7023	
Chemical compound, drug	DiI (1,1′-dioctadecyl-3,3,3′, 3′-tetramethylindocarbocyanine perchlorate)	Invitrogen	D282	
Chemical compound, drug	DiD′ solid; DiIC**_18_**(5) solid (1,1′-dioctadecyl-3,3,3′,3′- tetramethylindodicarbocyanine, 4-chlorobenzenesulfonate salt)	Invitrogen	D7757	
Chemical compound, drug	Dulbecco’s Modified Eagle Medium (DMEM)	Thermo Fisher Scientific	11965092	
Chemical compound, drug	Penicillin–Streptomycin	Thermo Fisher Scientific	15140122	
Chemical compound, drug	HEPES-NaOH	Homemade		
Chemical compound, drug	DMEM FluoroBrite	Thermo Fisher Scientific	A1896701	
Chemical compound, drug	FBS	Merck	F7524	
Chemical compound, drug	Rat serum	Janvier Labs	Labs Rat Serum Sprague Dawley RjHan SD (male only)	
Chemical compound, drug	Corn oil	Sigma-Aldrich	C8267	
Chemical compound, drug	4-Hydroxytamoxifen	Merck	H6278	
Software, algorithm	Fiji-ImageJ	Schindelin et al., 2012	RRID:SCR_002285	
Software, algorithm	ITK-SNAP (version 3.8.0)	Yushkevich et al., 2006	RRID:SCR_017341	
Software, algorithm	MeshLab (version 2020.06)	Cignoni et al., 2008.	RRID:SCR_027065	
Software, algorithm	Imaris (version 9.5)	Oxford Instruments/Bitplane	RRID:SCR_007370	
Software, algorithm	IBM SPSS Statistics	IBM Corp	RRID:SCR_016479	
Software, algorithm	MATLAB (version 2023.b)	The MathWorks, Inc	RRID:SCR_001622	
Software, algorithm	Paraview	Kitware, Inc	RRID:SCR_002516	

### Mouse strains

Animals were handled in accordance with CNIC Ethics Committee, Spanish laws and the EU Directive 2010/63/EU for the use of animals in research. All mouse experiments were approved by the CNIC and Universidad Autónoma de Madrid Committees for ‘Ética y Bienestar Animal’ and the area of ‘Protección Animal’ of the Community of Madrid with reference PROEX 220/15. For this study, mice were maintained on mixed C57Bl/6 or CD1 background. We used the following mouse lines, which were genotyped by PCR following the original study protocols. Male and female mice of more than 8 weeks of age were used for mating.

### Embryo culture and live imaging

Live-imaging procedures were performed following the protocol described by Sendra et al., 2022. Mouse embryos (E6.5–E7.5) were collected and dissected in dissection medium consisting of DMEM supplemented with 10% fetal bovine serum, 25 mM HEPES–NaOH (pH 7.2), and penicillin–streptomycin (50 µg/ml each). Embryos were cultured in a medium composed of 50% Janvier Labs Rat Serum (Sprague Dawley RjHan SD, male only) and 50% DMEM FluoroBrite (Thermo Fisher Scientific, A1896701) at 37°C under 7% CO_2_. Two-photon live imaging was performed on a Zeiss LSM780 microscope equipped with a 20×objective (NA = 1) and a MaiTai laser tuned to 980 nm for two-channel acquisition. Fluorescence signals were detected using non-descanned detectors with cyan-yellow (BP450–500/BP520–560), green-red (BP500–520/BP570–610), and yellow-red (BP520–560/BP645–710) filter sets. Image acquisition was controlled using Zen software (Zeiss) with an output power of 250 mW, a pixel dwell time of 14.8 µs, line averaging of two, and an image size of 1024 × 1024 pixels.

### Cell tracking with TAT-Cre microinjection and dye microinjection

Embryos at stages E6.5–E7.5 were dissected and cultured as described above using the same dissection and culture media. For microinjection experiments, embryos were maintained in a hypoxic chamber incubator at 37°C with 5% O_2_ and 7% CO_2_. Embryos were microinjected with TAT-Cre recombinase or fluorescent dyes as previously described (Sendra et al., 2023). Microinjection needles were prepared with a 2-µm gauge and inserted into the anterior side of the embryo until penetrating the endodermal layer, using specified pressure conditions. The embryos were handled and positioned carefully, ensuring that the anterior and posterior sides were oriented accordingly during the procedure to achieve successful microinjections.

We utilized mouse embryos that carried both the reporter genes ROSA26CAG–TdTomato (R26RtdTomato) and ROSA26CAG–EGFP (R26REGFP) in transheterozygosis. We fixed, imaged, and annotated fluorescent cells within anatomical regions. ‘Clusters’ were defined as groups of cells (either Tomato or GFP) originating from a single TAT-Cre injection. For dye microinjections, mouse embryos were labeled by injection of a lipophilic carbocyanine, DiI (1,1′-dioctadecyl-3,3,3′,3′-tetramethylindocarbocyanine perchlorate) and DiD (1,1′-dioctadecyl-3,3,3′,3′-tetramethylindodicarbocyanine), as described previously (Domínguez et al., 2012, Franco et al., 2001). Subsequently, embryos were incubated in increasing sucrose concentrations (10%, 20%, and 30%) for tissue cryoprotection, embedded, and cryosectioned using a cryostat. Images were acquired by confocal microscopy.

### Genetic cell labeling

To visualize cardiac tissue and achieve fluorescent contrast for motion estimation and cell tracking, embryos expressed fluorescent reporters under the control of several commonly used cardiac or mesodermal drivers. These included Nkx2.5-GFP and Nkx2.5-Cre lines to label myocardial tissue, Mesp1-Cre to label mesodermal derivatives, and Islet1-Cre to label a population enriched in SHF progenitors. We note that Islet1-Cre activity is not restricted to the SHF and includes additional cardiac progenitor populations, including subsets of left ventricular precursors. However, this does not affect the analyses presented here, as lineage identity is not used for biological interpretation.

Fluorescent labeling was used solely to create sufficient signal contrast to follow tissue motion or to identify individual cells for validation of the motion estimation algorithm. The computational framework does not rely on tracking specific cell types, nor does it assume lineage specificity. Instead, fluorescence serves as a generic marker of tissue continuity and motion, allowing validation of the image-based registration independently of the genetic driver used.

For sparse cell labeling, embryos carried a ubiquitously expressed CreERT2 allele (Polr2a-CreERT2, RERT) in combination with Rosa26 reporter alleles. Recombination was induced by low-dose tamoxifen administration to pregnant females, resulting in stochastic and sparse labeling of cells. Tamoxifen was prepared by dissolving 4-hydroxytamoxifen (Sigma) at 1 mg/ml in corn oil with ethanol as an intermediate solvent, followed by sonication. A single 0,2 mg intraperitoneal injection was administered at E6.5. Sparsely labeled cells are used exclusively as fiducial markers to provide ground-truth trajectories for validating the accuracy of the deformation fields computed from image registration. Labeling efficiency and spatial distribution were therefore assessed only to ensure sufficient numbers of isolated cells for validation purposes, rather than to achieve comprehensive or lineage-restricted coverage.

### Computational workflow

The computational workflow is detailed in Raiola et al., 2025. Here, we describe only the additional analysis introduced in this study that was not included in the methodological paper.

### Strain Agreement Index

We defined the stretch direction as the eigenvector related to the maximum eigenvalue. To better understand the stretching directions at the tissue level, we introduced an additional parameter called the Strain Agreement Index (*φ*). This parameter identified regions of the heart that stretched in the same direction, discriminating them from those with chaotic or discordant directions. *φ* was calculated as the percentage of directions that were in concordance in a certain neighborhood. The choice of the neighborhood size, corresponding to six to seven cells (approximately 20 pixels), was defined experimentally to achieve a balance between local resolution and robustness. This scale was small enough to allow the tissue to be computationally flattened, avoiding artifacts from folded regions, while larger neighborhoods would have reduced local sensitivity. Conversely, smaller neighborhoods would have produced fragmented, salt-and-pepper patterns lacking generalization.

We evaluated the *φ* index for each face considering the stretching in this neighborhood. As a first step, we projected the selected stretch vectors onto a plane identified by the centroids of the selected faces, using the *fitPlane* and *projLineOnPlane* functions of the geom3d library (http://github.com/mattools/matGeom/, Legland et al., 2026). In the second step, we calculated the angle formed by each stretch vector with the remaining vectors. We then evaluated the Agreement Index as the percentage of vectors whose angle diverged from ±10°. The percentage value, ranging between 0 and 100%, was converted into a color code and plotted for each mesh.

### Software

We recommend opening the 3D mesh with the open-source software ‘MeshLab’ (https://www.meshlab.net/). In ParaView (https://www.paraview.org), you can plot the MaxStrain vectors. Once the .vtk file is imported, the vectors can be visualized using the Glyph representation. It is recommended to display the vectors as lines for better visual interpretation.

To view and use the in silico Fate Map, the .ims data needs to be imported into Imaris Viewer (https://imaris.oxinst.com/imaris-viewer), the free version of the Imaris visualization software.

### Materials availability

This study did not generate new unique reagents.

## Data Availability

All image data generated during this study are publicly available in Mendeley Data at https://doi.org/10.17632/nd3kmj3cnx.1. The computational model is openly accessible at https://github.com/MorRaiola/BarrelHeartModel (copy archived at Raiola, 2026). The following datasets were generated: RaiolaM TorresM 2025Quantitative computerized analysis demonstrates strongly compartmentalized tissue deformation patterns underlying mammalian heart tube formationMendeley Data10.17632/nd3kmj3cnx.1PMC1339108242478151 RaiolaM TorresM 2025A method for analysing tissue motion and deformation during mammalian cardiogenesis. Raiola et al.2025bMendeley Data10.17632/54gbvnsgnp.1PMC1254889541091733

## Additional files

Supplementary file 1. Dataset of TAT-Cre microinjection and cell dye microinjection. eCre(20h) image was captured with Flash procedure (Messal et al., 2021).

Supplementary file 2. Dataset of cell dye microinjection experiments reporting labeling information for the right and left sides of the heart tube (HT). Embryos e1–e3 were used to measure D1, computed as a geodesic distance. Embryos e4 and e5 were used to measure D2, computed as a Euclidean point-to-point distance between fixed landmarks. Both the landmarks and the ROI are available in the Mendeley dataset (Figure 5). Fold changes are reported between t(0) and t(end) over an approximately 10-hr *time window.*

MDAR checklist

## References

[bib1] Buckingham M, Meilhac S, Zaffran S (2005). Building the mammalian heart from two sources of myocardial cells. Nature Reviews. Genetics.

[bib2] Cai CL, Liang X, Shi Y, Chu PH, Pfaff SL, Chen J, Evans S (2003). Isl1 identifies a cardiac progenitor population that proliferates prior to differentiation and contributes a majority of cells to the heart. Developmental Cell.

[bib3] Cignoni P, Callieri M, Corsini M, Dellepiane M, Ganovelli F, Ranzuglia G (2008). MeshLab: An open-source mesh processing tool.

[bib4] de Boer BA, van den Berg G, de Boer PAJ, Moorman AFM, Ruijter JM (2012). Growth of the developing mouse heart: an interactive qualitative and quantitative 3D atlas. Developmental Biology.

[bib5] Desgrange A, Le Garrec JF, Bernheim S, Bønnelykke TH, Meilhac SM (2020). Transient nodal signaling in left precursors coordinates opposed asymmetries shaping the heart loop. Developmental Cell.

[bib6] Domínguez JN, Meilhac SM, Bland YS, Buckingham ME, Brown NA (2012). Asymmetric fate of the posterior part of the second heart field results in unexpected left/right contributions to both poles of the heart. Circulation Research.

[bib7] Dominguez MH, Krup AL, Muncie JM, Bruneau BG (2023). Graded mesoderm assembly governs cell fate and morphogenesis of the early mammalian heart. Cell.

[bib8] Ebrahimi N, Osanlouy M, Bradley CP, Kubke MF, Gerneke DA, Hunter PJ (2022). A method for investigating spatiotemporal growth patterns at cell and tissue levels during C-looping in the embryonic chick heart. iScience.

[bib9] Esteban I, Schmidt P, Desgrange A, Raiola M, Temiño S, Meilhac SM, Kobbelt L, Torres M (2022). Pseudodynamic analysis of heart tube formation in the mouse reveals strong regional variability and early left-right asymmetry. Nature Cardiovascular Research.

[bib10] Franco D, Kelly R, Moorman AFM, Lamers WH, Buckingham M, Brown NA (2001). MLC3F transgene expression in iv mutant mice reveals the importance of left-right signalling pathways for the acquisition of left and right atrial but not ventricular compartment identity. Developmental Dynamics.

[bib11] Gu B, Posfai E, Rossant J (2018). Efficient generation of targeted large insertions by microinjection into two-cell-stage mouse embryos. Nature Biotechnology.

[bib12] Guerra C, Mijimolle N, Dhawahir A, Dubus P, Barradas M, Serrano M, Campuzano V, Barbacid M (2003). Tumor induction by an endogenous K-ras oncogene is highly dependent on cellular context. Cancer Cell.

[bib13] Ivanovitch K, Temiño S, Torres M (2017). Live imaging of heart tube development in mouse reveals alternating phases of cardiac differentiation and morphogenesis. eLife.

[bib14] Kawahira N, Ohtsuka D, Kida N, Hironaka KI, Morishita Y (2020). Quantitative analysis of 3D tissue deformation reveals key cellular mechanism associated with initial heart looping. Cell Reports.

[bib15] Kelly RG, Buckingham ME, Moorman AF (2014). Heart fields and cardiac morphogenesis. Cold Spring Harbor Perspectives in Medicine.

[bib16] Kidokoro H, Okabe M, Tamura K (2008). Time-lapse analysis reveals local asymmetrical changes in C-looping heart tube. Developmental Dynamics.

[bib17] Le Garrec JF, Ragni CV, Pop S, Dufour A, Olivo-Marin JC, Buckingham ME, Meilhac SM (2013). Quantitative analysis of polarity in 3D reveals local cell coordination in the embryonic mouse heart. Development.

[bib18] Le Garrec JF, Domínguez JN, Desgrange A, Ivanovitch KD, Raphaël E, Bangham JA, Torres M, Coen E, Mohun TJ, Meilhac SM (2017). A predictive model of asymmetric morphogenesis from 3D reconstructions of mouse heart looping dynamics. eLife.

[bib19] Legland D, Carbajal JP, Mikola RG, Schappler M, Silva GF (2026). Github.

[bib20] Lescroart F, Chabab S, Lin X, Rulands S, Paulissen C, Rodolosse A, Auer H, Achouri Y, Dubois C, Bondue A, Simons BD, Blanpain C (2014). Early lineage restriction in temporally distinct populations of Mesp1 progenitors during mammalian heart development. Nature Cell Biology.

[bib21] Madisen L, Zwingman TA, Sunkin SM, Oh SW, Zariwala HA, Gu H, Ng LL, Palmiter RD, Hawrylycz MJ, Jones AR, Lein ES, Zeng H (2010). A robust and high-throughput Cre reporting and characterization system for the whole mouse brain. Nature Neuroscience.

[bib22] Meilhac SM, Esner M, Kerszberg M, Moss JE, Buckingham ME (2004). Oriented clonal cell growth in the developing mouse myocardium underlies cardiac morphogenesis. The Journal of Cell Biology.

[bib23] Meilhac SM, Lescroart F, Blanpain C, Buckingham ME (2014). Cardiac cell lineages that form the heart. Cold Spring Harbor Perspectives in Medicine.

[bib24] Messal HA, Almagro J, Zaw Thin M, Tedeschi A, Ciccarelli A, Blackie L, Anderson KI, Miguel-Aliaga I, van Rheenen J, Behrens A (2021). Antigen retrieval and clearing for whole-organ immunofluorescence by FLASH. Nature Protocols.

[bib25] Moorman AFM, Christoffels VM (2003). Cardiac chamber formation: development, genes, and evolution. Physiological Reviews.

[bib26] Myronenko A, Song X (2010). Intensity-based image registration by minimizing residual complexity. IEEE Transactions on Medical Imaging.

[bib27] Nowotschin S, Xenopoulos P, Schrode N, Hadjantonakis AK (2013). A bright single-cell resolution live imaging reporter of Notch signaling in the mouse. BMC Developmental Biology.

[bib28] Raiola M, Sendra M, Torres M (2023). Imaging approaches and the quantitative analysis of heart development. Journal of Cardiovascular Development and Disease.

[bib29] Raiola M, Esteban I, Ivanovitch K, Sendra M, Torres M (2025). A method for analysing tissue motion and deformation during mammalian organogenesis. bioRxiv.

[bib30] Raiola M (2026). https://archive.softwareheritage.org/swh:1:dir:aa5972a48dc99bf1fe615f9969f7bc37f04c8d78;origin=https://github.com/MorRaiola/BarrelHeartModel;visit=swh:1:snp:6c5fdeab2e263532d1ec98067f3c04d68f2849fa;anchor=swh:1:rev:aad22e69b7b74b26080ae3df0448aad9ce7c2135.

[bib31] Schindelin J, Arganda-Carreras I, Frise E, Kaynig V, Longair M, Pietzsch T, Preibisch S, Rueden C, Saalfeld S, Schmid B, Tinevez JY, White DJ, Hartenstein V, Eliceiri K, Tomancak P, Cardona A (2012). Fiji: an open-source platform for biological-image analysis. Nature Methods.

[bib32] Sendra M, Mañes J, Domínguez JN, Torres M (2022). Live imaging of early cardiac progenitors in the mouse embryo. Journal of Visualized Experiments.

[bib33] Sendra M, de Dios Hourcade J, Temiño S, Sarabia AJ, Ocaña OH, Domínguez JN, Torres M (2023). Cre recombinase microinjection for single-cell tracing and localised gene targeting. Development.

[bib34] Sendra M, McDole K, de Dios Hourcade J, Temiño S, Raiola M, Guignard L, Keller PJ, Domínguez JN, Torres M (2025). Myocardium and endocardium of the early mammalian heart tube arise from independent multipotent lineages specified at the primitive streak. Developmental Cell.

[bib35] Shi Y, Yao J, Young JM, Fee JA, Perucchio R, Taber LA (2014). Bending and twisting the embryonic heart: a computational model for c-looping based on realistic geometry. Frontiers in Physiology.

[bib36] Sousa VH, Miyoshi G, Hjerling-Leffler J, Karayannis T, Fishell G (2009). Characterization of Nkx6-2-derived neocortical interneuron lineages. Cerebral Cortex.

[bib37] Stanley EG, Biben C, Elefanty A, Barnett L, Koentgen F, Robb L, Harvey RP (2004). Efficient Cre-mediated deletion in cardiac progenitor cells conferred by a 3’UTR-ires-Cre allele of the homeobox gene Nkx2-5. The International Journal of Developmental Biology.

[bib38] von Gise A, Lin Z, Schlegelmilch K, Honor LB, Pan GM, Buck JN, Ma Q, Ishiwata T, Zhou B, Camargo FD, Pu WT (2012). YAP1, the nuclear target of Hippo signaling, stimulates heart growth through cardiomyocyte proliferation but not hypertrophy. PNAS.

[bib39] Wu SM, Fujiwara Y, Cibulsky SM, Clapham DE, Lien CL, Schultheiss TM, Orkin SH (2006). Developmental origin of a bipotential myocardial and smooth muscle cell precursor in the mammalian heart. Cell.

[bib40] Yushkevich PA, Piven J, Hazlett HC, Smith RG, Ho S, Gee JC, Gerig G (2006). User-guided 3D active contour segmentation of anatomical structures: significantly improved efficiency and reliability. NeuroImage.

