# Supplementary material for: Quantitative computerized analysis demonstrates strongly compartmentalized tissue deformation patterns underlying mammalian heart tube formation
Source: eLife. 2026 Jul 21;14:RP108559. doi: 10.7554/eLife.108559 (PMC13391082; doi:10.7554/eLife.108559)
Supplement: Supplementary file 1. — eCre(20h) image was captured with Flash procedure (Messal et al., 2021). [file elife-108559-supp1.docx]

| Embryo | Genotype | VoxelRes [μm] |
| --- | --- | --- |
| eCre(0h) | R26REGFP ; Rosa26Rtdtomato | 1.136-1.136-2.99 |
| eCre(20h) | Dapi ; R26REGFP ; Rosa26Rtdtomato ; anti-M20(1:100) | 0.757-0.757-1.04 |
| eDye(0h) | Nkx2.5GFP ; Polr2a–CreERT2 (RERT) ; Rosa26Rtdtomato | 1.636-1.636-2.99 |
| eDye(15h) | Nkx2.5GFP ; Polr2a–CreERT2 (RERT) ; Rosa26Rtdtomato | 0.734-0.734-6.00 |
