# Supplementary material for: Quantitative computerized analysis demonstrates strongly compartmentalized tissue deformation patterns underlying mammalian heart tube formation
Source: eLife. 2026 Jul 21;14:RP108559. doi: 10.7554/eLife.108559 (PMC13391082; doi:10.7554/eLife.108559)
Supplement: Supplementary file 2. — Embryos e1–e3 were used to measure D1, computed as a geodesic distance. Embryos e4 and e5 were used to measure D2, computed as a Euclidean point-to-point distance between fixed landmarks. Both the landmarks and the ROI are available in the Mendeley dataset (Figure 5). Fold changes are reported between t(0) and t(end) over an approximately 10-hr time window. [file elife-108559-supp2.docx]

| Embryo | Labeling | | Fold change |
| --- | --- | --- | --- |
|  | Left | Right |  |
| e1_D1 | diI | diD | 0.23x |
| e2_D1 | diI | diD | 0.51x |
| e3_D1 | diI | diD | 0.77x |
| e4_D2 | diI | diD | 0.13x |
| e5_D2 | diI | diD | 0.29x |
| e6_D2 | diD | diI | 0.40x |
