# Supplementary material for: Quantitative computerized analysis demonstrates strongly compartmentalized tissue deformation patterns underlying mammalian heart tube formation
Source: eLife. 2026 Jul 21;14:RP108559. doi: 10.7554/eLife.108559 (PMC13391082; doi:10.7554/eLife.108559)
Supplement: Peer review history

# Editor report: eLife Assessment

**DOI:** 10.7554/eLife.108559.3.sa0

**Authors:** Robert G Kelly

**Affiliations:** Aix-Marseille Université France

**Keywords:** Compelling, Fundamental

This **fundamental** work substantially advances our understanding of tissue deformation and growth patterns during the earliest stages of mammalian heart development. One of the strengths of the work is the **compelling** quantitative approach to analyzing time-lapse imaging data using an original computational pipeline, which goes beyond the current state of the art and provides new insights into heart tube formation. Overall, this rigorous study will be of broad interest to computational and developmental biologists studying tissue dynamics.

# Referee report: Reviewer #1 (Public review)

**DOI:** 10.7554/eLife.108559.3.sa1

[Editors' note: this version has been assessed by the Reviewing Editor without further input from the original reviewers. The authors have addressed all the comments raised in the previous round of review. The revised manuscript includes new labeling experiments revealing boundary compression at the cardiac poles consistent with the authors predicted dynamic model of heart tube formation.]

Summary:

The study by Raiola et al. conducted a quantitative analysis of tissue deformation during the formation of the primitive heart tube from the cardiac crescent in mouse embryos. Using the tools developed to analyze growth, anisotropy, strain, and cell fate from time-lapse imaging data of mouse embryos, the authors elucidated the compartmentalization of tissue deformation during heart tube formation and ventricular expansion. This paper describes how each region of the cardiac tissue changes to form the heart tube and ventricular chamber, contributing to our understanding of the earliest stages of cardiac development.

In order to understand tissue deformation in cardiac formation, it is commendable that the authors effectively utilized time-lapse imaging data, a data pipeline, and in silico fate mapping. The study clarifies the compartmentalization of tissue deformation by integrating growth, anisotropy, and strain patterns in each region of the heart.

# Referee report: Reviewer #2 (Public review)

**DOI:** 10.7554/eLife.108559.3.sa2

The authors address an important challenge in developmental biology: the quantitative description of tissue deformation during organogenesis. They have developed a new pipeline to quantify early heart tube morphogenesis in the mouse, with cellular resolution. They adopt an elegant approach by integrating multiple 3D time-lapse datasets into a dynamic atlas of cardiac morphogenesis in order to compute spatio-temporal deformation patterns. The main findings highlight a strong compartmentalization of cell behaviors, with tissue growth and anisotropy exhibiting complementary and spatially segregated patterns. Using these data, the authors developed an in-silico fate mapping tool to interrogate cell displacement within the myocardium. This virtual model provides new mechanistic insights into how the bilateral cardiac primordia converge and transform into a three-dimensional heart tube. The authors identify "belt-like" constraints at the arterial and venous poles that prevent tissue expansion and thus shape the ventricular barrel morphology.

The computational framework is highly innovative and impressive, providing an unprecedented 3D model of tissue deformation during heart morphogenesis. It also opens avenues for testing hypotheses regarding tissue growth and the forces that cause cell motion.

Overall, this carefully performed study provides a new model for exploring tissue deformation during organogenesis and will be of broad interest to computational and developmental biologists.

# Referee report: Reviewer #3 (Public review)

**DOI:** 10.7554/eLife.108559.3.sa3

Summary:

The manuscript by Raiola and colleagues entitled "Quantitative computerized analysis demonstrates strongly compartmentalized tissue deformation patterns underlying mammalian heart tube formation" takes a highly quantitative approach to interrogating the earliest stages of cardiogenesis (12 hours, from early cardiac crescent to early heart tube) in a new and innovative way. The paper presents a new computational framework to help identify both regional and temporal patterns of tissue deformation at cellular resolution. The method is applied to live embryo imaging data (newly generated and from the group's previous pioneering work). In the initial setup, the new model was applied directly to raw time-lapse data, and the results were compared to actual cell tracks identified manually, showing close correlations of the model with the manual tracking. Next, they integrated spatial and temporal information from different embryos to generate a new model for tissue movement, driven by parameters such as tissue growth and anisotropy. Key findings from their model suggest that there are distinct compartments of tissue deformation patterns as the bilateral cardiac crescent develops into the linear heart tube, and that the ventricular chamber forms by a defined expansion pattern, as a 'hemi-barrel shape', with the arterial and venous poles (IFT and OFT) acting as the harnessing belts constraining the expansion of the chamber further. Lastly, the model is tested for its ability to predict future residence of cardiac crescent cells in the heart tube, which it seems to be able to do successfully based on fate tracking validation experiments.

The manuscript provides an exceptionally careful analysis of a critical stage during heart development - that of the earliest stages of morphogenesis, when the heart forms its first tube and chamber structures. While numerous studies have interrogated this stage of heart development, few studies have performed time-lapse imaging, and, to my knowledge, no other report has performed such in in-depth quantitative analysis and modeling of this complex process. The computational model applied to normal heart development of the myocardium (labelled by Nkx2-5) has revealed multiple new and interesting concepts, such as the distinct compartments of tissue deformation patterns and the growth trajectories of the emerging ventricle. The fact that the model operates at cellular resolution and over a nearly continuous time period of approximately 12 hours allows for unprecedented depth of the analysis in a largely unbiased manner. Going forward, one can imagine such models revealing additional information on these processes, performing analyses of subpopulations that form the heart, and maybe most importantly, applying the model to various perturbation models (genetic or otherwise). The manuscript is very well written, and the data display is accessible and transparent.

No major weaknesses are noted with the study. It would have been very exciting to see the model applied to any kind of perturbation, for example, a left-right defect model, or a model with compromised cardiac progenitor populations. However, the amount of live imaging required for such analyses renders this out of scope for the current study.

# Author comment: Author response

**DOI:** 10.7554/eLife.108559.3.sa4

**Authors:** Morena Raiola, Miquel Sendra Sendra, Jorge N Domínguez, Miguel Torres

**Affiliations:** Spanish National Centre for Cardiovascular Research Madrid Spain; Institut de Biologie du Développement Marseille Marseille France; Universidad de Jaén Jaén Spain; Spanish National Centre for Cardiovascular Research Madrid Spain

The following is the authors’ response to the original reviews.

**Reviewer #1 (Public review):**

Summary:

The study by Raiola et al. conducted a quantitative analysis of tissue deformation during the formation of the primitive heart tube from the cardiac crescent in mouse embryos. Using the tools developed to analyze growth, anisotropy, strain, and cell fate from timelapse imaging data of mouse embryos, the authors elucidated the compartmentalization of tissue deformation during heart tube formation and ventricular expansion. This paper describes how each region of the cardiac tissue changes to form the heart tube and ventricular chamber, contributing to our understanding of the earliest stages of cardiac development.

Strengths:

In order to understand tissue deformation in cardiac formation, it is commendable that the authors effectively utilized time-lapse imaging data, a data pipeline, and in silico fate mapping.

The study clarifies the compartmentalization of tissue deformation by integrating growth, anisotropy, and strain patterns in each region of the heart.

Weaknesses:

The significance of the compartmentalization of tissue deformation for the heart tube formation remains unclear.

While it is obvious that the patterns of deformation should be relevant to model the cardiac crescent into the primitive cardiac tube, we do not provide direct evidence that changing these patterns affects heart tube formation. In this sense, the Reviewer is correct and this is a limitation of the study.

**Reviewer #1 (Recommendations for the authors):**

(1) It is interesting that growth rate and anisotropy are anticorrelated. However, the functional significance of this anticorrelation in heart formation remains unclear. It may be worthwhile to analyse the importance of the relationship between the two by adding inhibitors to cultured embryos or using mutant mouse models.

We appreciate this thoughtful suggestion and agree that such experimental approaches, involving inhibitors or mutant mouse models, could provide powerful validation of the proposed relationship. However, generating the appropriate lines and performing the necessary quantifications would represent a substantial effort that extends beyond the scope of the current study. Our focus here is to establish the correlation and its potential implications, leaving these more in-depth mechanistic investigations for future work.

(2) The authors claim to have analysed tissue deformation at the cellular level. Although cell labelling of specific regions using Tat-Cre and DiI injection and tracking of their fate have been performed, this still gives the impression of tissue-level analysis. An analysis "at the cellular level" would be expected to describe morphology, proliferation, polarity, etc., at the single-cell to multi-cell level.

We thank the reviewer for the comment. Our analysis does not involve single-cell characterization (e.g., morphology, proliferation, polarity) but focuses on quantifying tissue motion. The motion extracted from the images achieves cellular-level precision, as demonstrated by testing the registration algorithm and validating it against cellular tracking experiments. The accuracy of the method is therefore at the cellular scale. The goal of our study is to describe tissue dynamics during heart development, not to perform detailed cellular analyses. The novelty of our approach is that it enables tissuescale quantification in developmental mouse heart imaging, where cell density and image resolution make automated single-cell tracking unfeasible. By using fluorescence labelling as markers, we obtain cellular-level accuracy in tissue motion quantification.

(3) It is stated that cardiomyocytes, cardiac mesodermal cells, and SHF cells were labeled with Nkx2.5-GFP/Nkx2.5-Cre, Mesp1-Cre, and Islet1-Cre, respectively; however, the results of the labeling using these mice are not presented, and the reason for using different mouse strains is not apparent. Information on these mouse strains is missing in the Materials & Methods section. In particular, attention must be paid to mice of the same name but different strains. Islet1-Cre mice are not SHF-specific and exhibit activity in part of the left ventricular progenitors. Sparse labeling induced by low-dose tamoxifen administration is also unclear regarding the timing and concentration of tamoxifen administration. The authors should provide data on labeling efficiency and region, and also discuss the usefulness of analyses using different mouse strains.

We thank the reviewer for raising these important points. In this study, the use of different mouse strains was not driven by a biological comparison or lineage-specific analysis, but by the availability of high-quality cardiac imaging datasets generated in the laboratory. The primary goal of this work is methodological: to demonstrate that developmental cardiac imaging data can be reused within an engineering framework to quantify tissue deformation. For this purpose, we do not track individual cells but instead use fluorescence labelling as a versatile strategy to follow tissue motion without requiring a strain- or lineage-specific labelling.

We acknowledge that Islet1-Cre mice are not SHF-specific and exhibit activity in part of the left ventricular progenitors. However, this limitation does not affect our analysis, as the specificity of the labelled cells is not used in the image processing or deformation quantification pipeline.

Regarding tamoxifen, we clarified the dosage and administration in the revised Experimental workflow section. Importantly, tamoxifen treatment does not influence the proposed image analysis framework, since labelled cells are employed solely as fiducial references to provide ground-truth validation of the tissue motion estimated from image registration.

We made these points clearer in the Results section and in Materials and Methods.

(4) It is noteworthy that the authors have utilized many new analytical methods that they have developed. In the analysis presented in this paper, it is understandable that the methods described in another paper by the authors (Raiola M et al., 2025) are utilized; however, it is important to note that this causes some overlap. It is necessary to clearly distinguish and describe whether the novelty of the methods is based on those developed in this paper or those described in the paper by Raiola M et al. (2025).

We thank the reviewer for this important observation. We agree that it is essential to clearly separate the methodological developments reported in Raiola M et al. (2025) from the present work. As described in Raiola M et al. (2025), the methods have already been fully developed and validated. In this paper, our focus is to apply these approaches to cardiac development in order to generate and describe the new biological insights. In the revised version, we made this distinction more explicit in the Results and Discussion, highlighting the methodological continuity with our previous work and the biological contribution of the present study.

Minor points

(1) In Figure 4, the labels and legends for A, A', B, and B' are reversed. Similar colours are used for C through F, making it difficult to distinguish between them.

We thank the reviewer for noticing this error. We have corrected it.

(2) In Figure 5, the labels start with B.

We thank the reviewer for noticing this error. We have corrected the labels in Figure 5.

**Reviewer #2 (Public review):**

The authors address an important challenge in developmental biology: the quantitative description of tissue deformation during organogenesis. They have developed a new pipeline to quantify early heart tube morphogenesis in the mouse, with cellular resolution. They adopt an elegant approach by integrating multiple 3D time-lapse datasets into a dynamic atlas of cardiac morphogenesis in order to compute spatio-temporal deformation patterns. The main findings highlight a strong compartmentalization of cell behaviors, with tissue growth and anisotropy exhibiting complementary and spatially segregated patterns. Using these data, the authors developed an in-silico fate mapping tool to interrogate cell displacement within the myocardium. This virtual model provides new mechanistic insights into how the bilateral cardiac primordia converge and transform into a three-dimensional heart tube. The authors identify "belt-like" constraints at the arterial and venous poles that prevent tissue expansion and thus shape the ventricular barrel morphology.

The computational framework is highly innovative and impressive, providing an unprecedented 3D model of tissue deformation during heart morphogenesis. It also opens avenues for testing hypotheses regarding tissue growth and the forces that cause cell motion. However, the proposed model of ventricular chamber formation with the two constraining belts remains hypothetical, lacking biological validation and requiring strengthening or modulation.

Overall, this carefully performed study provides a new model for exploring tissue deformation during organogenesis and will be of broad interest to computational and developmental biologists.

We agree with the Reviewer on the limitations of the proposed model due to limited experimental validation. In the revised version of the manuscript we provide further experimental evidence that strengthens the biological validation of the proposed barrel model with two transversal “belts” generating the barrel shape of the primitive ventricle.

**Reviewer #2 (Recommendations for the authors):**

(1) The study proposes a new model of heart morphogenesis by identifying two regions of tissue contraction at the arterial and venous poles. Although the fate map tool has been validated using two ex vivo approaches (DyeI microinjection and TAT-Cre genetic labelling), the conclusions regarding the two belts still need to be demonstrated using in vivo/ex vivo experiments and quantification of cell movements.

We thank the reviewer for this important suggestion. We agree that experimental validation of the two contraction belts is essential to strengthen the conclusions of the study. In the revised manuscript, we have addressed this point by adding new experimental data directly supporting the existence and dynamics of both D1 and D2 contraction boundaries.

Specifically, we performed microinjection experiments in which four anchor points, two along D1 and two along D2, were labeled in living embryos and tracked after 10–14 hours (Figure 5C–E, Table S2). For D2, the Euclidean distance between the two anchor points was computed from multiphoton microscopy images acquired at t0 and tfinal (voxel size: 0.57 × 0.57 × 2.5–6.0 µm). In all three embryos analyzed, the D2 anchor points converged over time, with the segment retaining on average 0.27 ± 0.14 of its initial length (range: 0.13–0.40), confirming the lateral compression predicted by the model. For D1, the in-plane geodesic distance between anchor points was measured at t0 and after 10–14 hours. Given the difficulty of imaging the arterial pole at high resolution by whole-mount microscopy, cryosections were used for these measurements (pixel size: 0.65 × 0.65 × 0.042 µm). The D1 segment similarly underwent contraction, retaining on average 0.50 ± 0.22 of its initial length (range: 0.23–0.77). Together, these results provide direct experimental evidence that both boundaries undergo compression during heart tube formation, consistent with the contraction dynamics predicted by the virtual model and supporting the existence of the two belts described in the study.

We acknowledge that the quantitative values show variability across embryos, which reflects two main sources of uncertainty: (i) the exact position of microinjection along D1 and D2 could not be perfectly standardized; (ii) embryos were not staged at exactly stage 2 at t0 nor did they all reach exactly stage 8 at tend, introducing stage-dependent variability. The primary goal of this experiment was therefore not to precisely quantify compression rates, but to demonstrate that tissue contraction along both boundaries occurs in vivo, consistent with the barrel model predictions. The fact that contraction was observed in all six embryos analyzed, despite the inherent variability of the experimental setup, supports the robustness of this conclusion. These points have been discussed in the revised manuscript.

(2) The region labelled as OFT appears to correspond instead to the right ventricle primordium, as demonstrated previously by cell labelling of the anterior heart field (Zaffran et al., 2004, PMID: 15217909). The nomenclature should be corrected in the figures and the text. Alternatively, the term "arterial pole" may be useful.

We thank the reviewer for this observation. We aligned our nomenclature with the literature, correcting the labelling in figures and text.

(3) The integration of 12 different time-lapses into the model is very impressive. However, while the early stages (2 to 5) are very well covered, the number of replicates for the later stages is much lower. Figure S4 highlights variability between some of the samples, but this is not commented on in the results or the discussion. How does this impact the averaging of tissue deformation patterns and the subsequent model predictions? We thank the reviewer for this comment. We acknowledge that the number of specimens is lower and more variable at later stages. This limitation primarily arises from technical constraints associated with long time-lapse imaging. Because embryo positioning could not be actively tracked during growth, manual repositioning was required, and since embryo development proceeded overnight, maintaining perfect alignment throughout the acquisition was challenging. As a result, several embryos gradually drifted out of the imaging volume and had to be excluded due to incomplete coverage. In addition, at later stages the onset of uncoordinated and subsequently coordinated cardiomyocyte contractions introduces motion-related blurring, which further limits image quality at the acquisition frequency used. These technical limitations were already discussed in the context of the imaging methodology and Limitation and Future Directions section in Raiola et al. (2025).

As shown in Figure S4, variability between embryos is present and reflects natural biological diversity. Figure S4 also indicates that this variability is highly localized, whereas the regions identified as anticorrelated growth and anisotropy zones remain consistently preserved across embryos. The variability observed in Figure S4, we note that while inter-embryo variability is present, it mainly affects the magnitude of tissue deformation rather than the spatial pattern of deformation. As shown in the additional analyses presented in Figures S5 and S6, the overall organization of deformation, both in terms of growth and anisotropy, is consistently preserved among embryos within the same stage group, within the expected range of natural intra-embryonic variability.

Finally, regarding the in-silico fate map, our model was not constructed as a statistical average but as a descriptive framework obtained from the concatenation of selected representative embryos. Constructing a statistical model was not feasible due to the limited number of embryos at later stages and the frequent occurrence of incomplete datasets (e.g., randomly missing inflow or arterial pole regions). Under such conditions, only the left ventricular primordium and the inner curvature would have been consistently preserved, thereby limiting the analysis to a very restricted and less informative region. We emphasized these points more clearly in the revised Result section.

(4) Since the growth rate appears to be highly regionalized, could the authors provide a molecular mechanism for one of these growth patterns?

We thank the reviewer for this insightful suggestion. Although correlating growth patterns with specific molecular mechanisms would greatly enhance the study, such an effort necessitates extensive additional experimentation, including spatial transcriptomics and detailed molecular analyses. As this falls outside the scope of the present work, we have chosen not to incorporate molecular mechanism data in this manuscript, reserving it for future research.

(5) Could the model be used to predict new experimental outcomes? For example, could the author simulate a perturbation and validate it through in vivo experiments using mouse mutants?

We thank the reviewer for this interesting suggestion. At this stage, the model cannot be used to predict new experimental outcomes, as it was designed as a descriptive rather than a statistical or predictive framework. The predictive potential of the model, including the simulation of perturbations, was discussed in detail in Raiola et al. (2025), where this aspect was indicated as a direction for future work.

We clarified this more explicitly in the revised Results and Discussion sections.

Minor points

(1) The readership of eLife is diverse. The methodology and figures could be further annotated, and the axes (A/P, D/V, L/R) could be labelled in all figure panels.

We thank the reviewer for this helpful suggestion. We revised the figures to include clearer annotations and ensure that the axes (A/P, D/V, L/R) are consistently labelled across all panels.

(2) It is sometimes difficult to follow without reference to the companion paper. For example, machine learning is mentioned in the summary but is not described in this paper.

We thank the reviewer for this comment. We clarified in the revised manuscript that the staging system is machine learning-based, using morphometric features to align specimens over time, and indicate that full methodological details are provided in Raiola et al. (2025). This will help readers understand the approach while keeping the focus on the biological findings.

(3) The authors state the versatility of the model in the introduction, but this is not really addressed in the manuscript; please modulate.

We thank the reviewer for this feedback. We agree that the versatility of the model was not sufficiently demonstrated throughout the manuscript. In the revised version, we rephrased the Summary to ensure that our claims are aligned with the descriptive scope shown in the current study.

(4) The authors describe a rightward rotation of the ventricle in stage 9, which they relate to the arterial pole rotation described by Le Garrec et al., 2017. However, this event was reported to occur at E8.5f (which would be equivalent to stage 7). Please modulate or modify.

We thank the reviewer for this observation. Heart tube rotation is a gradual process that begins at earlier stages, including stage 7, depending on embryo developmental variability. In our study, using the Atlas-based framework described by Esteban et al. (2022), this rotation becomes clearly detectable and morphologically prominent at stage 9, as illustrated in Figure 6d of Esteban et al. At this stage, rightward rotation of the ventricle emerges as the dominant feature in terms of tissue deformation and associated growth patterns, providing a robust reference point to describe and quantify the process. Thus, the description of stage 9 does not indicate the initiation of ventricular rotation, but rather the stage at which the process is most evident and measurable. We moderated it into the revised manuscript to avoid potential ambiguity.

(5) Some rationales are missing. Why aren't all of the initial 16 time-lapses used for the cumulative deformation pattern analysis? Please explain the impact on the virtual fate mapping of using either labelling of cell clusters or cell continuums. Explain how the Strain Agreement Index neighborhood size (6-7 cells) was chosen, and whether the results are robust at other scales.

We thank the reviewer for raising these important points. We agree that this section requires clarification and will expand it in the revised Results and Discussion. Not all 16 time-lapses could be included in the cumulative deformation analysis, as this approach relies on concatenating individual embryos into the Atlas framework while preserving the largest possible overlap of tissue. A technical limitation of our recordings was the nonsystematic loss of cardiac tube extremities (inflow tract or arterial pole) due to embryo drift during acquisition. Consequently, several time-lapses provided incomplete tissue coverage and were excluded to avoid an inconsistent assessment of cumulative deformation. In fact, some regions of the tissue would have reflected the contribution of multiple embryos, whereas others would not. Moreover, the registration required to align anatomical regions across stages and embryos would have yielded inaccurate correspondences. For these reasons, we decided to exclude such cases. We commented on this point in more detail in the revised manuscript. For the Strain Agreement Index, the choice of a 6–7 cell neighbourhood size represented a balance between local resolution and robustness. This scale was small enough to allow the tissue to be computationally flattened, while larger neighbourhoods would have included folded regions and created artefacts during the flattening step. Conversely, smaller neighbourhoods would have produced fragmented, “salt-and-pepper” patterns lacking generalization. We commented on this point in more detail in the revised manuscript.

(6) Figure 5: The panels are mislabelled (B-C versus A-B).

We thank the reviewer for noticing this mistake. We have corrected the panel labels in Figure 5 to ensure consistency.

(7) Figure 5C: The red region in stage 2 within the IFT is missing.

We thank the reviewer for this observation. We have corrected Figure 5C accordingly.

(8) Typo in Figure 1 legend (p.5): "Our dataset includes multiple specimens raging from E7.75 to E8.25" - should be "ranging".

We thank the reviewer for pointing this out. We have corrected the typo in the Figure 1 legend.

(10) Figure S3 legend should state: "Deformation analysis for stage 7, stage 8, and stage 9."

We thank the reviewer for pointing this out. We have revised the Figure S3 legend accordingly.

**Reviewer #3 (Public review):**

Summary:

The manuscript by Raiola and colleagues entitled "Quantitative computerized analysis demonstrates strongly compartmentalized tissue deformation patterns underlying mammalian heart tube formation" takes a highly quantitative approach to interrogating the earliest stages of cardiogenesis (12 hours, from early cardiac crescent to early heart tube) in a new and innovative way. The paper presents a new computational framework to help identify both regional and temporal patterns of tissue deformation at cellular resolution. The method is applied to live embryo imaging data (newly generated and from the group's previous pioneering work). In the initial setup, the new model was applied directly to raw time-lapse data, and the results were compared to actual cell tracks identified manually, showing close correlations of the model with the manual tracking. Next, they integrated spatial and temporal information from different embryos to generate a new model for tissue movement, driven by parameters such as tissue growth and anisotropy. Key findings from their model suggest that there are distinct compartments of tissue deformation patterns as the bilateral cardiac crescent develops into the linear heart tube, and that the ventricular chamber forms by a defined expansion pattern, as a 'hemi-barrel shape', with the aterial and venous poles (IFT and OFT) acting as the harnessing belts constraining the expansion of the chamber further. Lastly, the model is tested for its ability to predict future residence of cardiac crescent cells in the heart tube, which it seems to be able to do successfully based on fate tracking validation experiments.

Strengths:

The manuscript provides an exceptionally careful analysis of a critical stage during heart development - that of the earliest stages of morphogenesis, when the heart forms its first tube and chamber structures. While numerous studies have interrogated this stage of heart development, few studies have performed time-lapse imaging, and, to my knowledge, no other report has performed such in in-depth quantitative analysis and modeling of this complex process. The computational model applied to normal heart development of the myocardium (labelled by Nkx2-5) has revealed multiple new and interesting concepts, such as the distinct compartments of tissue deformation patterns and the growth trajectories of the emerging ventricle. The fact that the model operates at cellular resolution and over a nearly continuous time period of approximately 12 hours allows for unprecedented depth of the analysis in a largely unbiased manner. Going forward, one can imagine such models revealing additional information on these processes, performing analyses of subpopulations that form the heart, and maybe most importantly, applying the model to various perturbation models (genetic or otherwise). The manuscript is very well written, and the data display is accessible and transparent.

Weaknesses:

No major weaknesses are noted with the study. It would have been very exciting to see the model applied to any kind of perturbation, for example, a left-right defect model, or a model with compromised cardiac progenitor populations. However, the amount of live imaging required for such analyses renders this out of scope for the current study.

We agree with the Reviewer on the relevance of applying this pipeline to mutant conditions. We are engaged on those experiments but they represent a major effort beyond the scope of this manuscript, as also indicated by the Reviewer.

**Reviewer #3 (Recommendations for the authors):**

(1) Application of the model to defective heart development:

While including perturbation models seems out of scope for the present work, some discussion on how the model might benefit our understanding of early cardiac defects, or any currently unknown mechanisms acting at this stage of development, could be included in the discussion of the manuscript. This would help highlight the enormous power that this new model could bring to understanding these critical steps during heart development, in a quantitative and unbiased manner.

We thank the reviewer for this insightful comment. Our approach is a deterministic, descriptive framework that integrates individual tissue motion into a common spatiotemporal Atlas, providing a quantitative description of early HT morphogenesis. The primary goal of this framework is to establish a robust baseline of normal HT development under wild-type conditions.

This baseline is essential for studying heart defects, as deviations from normal tissue motion and deformation patterns can reveal developmental defects like altered growth or aberrant morphogenetic trajectories. Currently, the limited number of embryos per developmental stage (typically 2-4) does not allow the construction of statistically robust inferential models. Nevertheless, by mapping all embryos into a unified reference system and providing quantitative descriptors of tissue motion, our framework already enables meaningful comparisons between normal and abnormal development.

We have clarified this point in the Discussion section.

(2) Confusion with Raiola et al., 2025:

The manuscript frequently references an accompanying manuscript, which is currently a preprint on bioRxiv. The relationship of these two papers is not clear from the description. Not only is the majority of the data shared between the reports, but some figures seem to overlap quite substantially. The methods state that "the computational workflow is detailed in Raiola et al 2025". Any clarification on this would be helpful.

We thank the reviewer for raising this important point and we appreciate the opportunity to clarify the relationship between the two manuscripts. The two studies indeed rely on the same underlying dataset; however, their aims and scope are fundamentally different. Raiola et al. (2025) is a purely methodological study, whose sole objective is to describe, validate, and benchmark a computational framework for spatiotemporal alignment, motion integration, and in-silico fate mapping. That work deliberately avoids biological interpretation, as the proposed approach is designed to be general and transferable to other organs or developmental systems.

In contrast, the present manuscript represents the biological application of this validated framework. Here, the computational model is used as a tool to extract, characterize, and interpret biologically meaningful information about early heart morphogenesis, including myocardial motion patterns, regional growth and anisotropy, and fate relationships, supported by experimental validation.

To avoid any ambiguity, we revised the Introduction and Materials and Methods to explicitly state this distinction and clarify why the methodological details are provided in Raiola et al. (2025), while the current manuscript focuses on biological insight rather than computational development.

(3) Additional point:

Concerning overlap with the authors' related manuscript in Bioarchive on the computational workflow: the number of specimens analysed should be noted without referral to the second manuscript (as currently mentioned in the figure legends). Is the "b" necessary when referring to the second manuscript?

We thank the reviewer for this suggestion. We included the number of specimens analysed directly in the revised manuscript to improve clarity for the reader. Regarding the citation format, the "b" in Raiola et al. (2025) is used to distinguish between two manuscripts from the same group published in the same year.
